# Study of Novel Peptides for Antimicrobial Protection in Solution and on Cotton Fabric

**DOI:** 10.3390/molecules27154770

**Published:** 2022-07-26

**Authors:** Petar Todorov, Stela Georgieva, Desislava Staneva, Petia Peneva, Petar Grozdanov, Ivanka Nikolova, Evgenia Vasileva-Tonkova, Ivo Grabchev

**Affiliations:** 1 Department of Organic Chemistry, University of Chemical Technology and Metallurgy, 1756 Sofia, Bulgaria; petenceto_2@abv.bg; 2 Department of Analytical Chemistry, University of Chemical Technology and Metallurgy, 1756 Sofia, Bulgaria; st.georgieva@uctm.edu; 3 Department of Textile and Leathers, University of Chemical Technology and Metallurgy, 1756 Sofia, Bulgaria; grabcheva@mail.bg; 4 The Stephan Angeloff Institute of Microbiology, Bulgarian Academy of Sciences, 1113 Sofia, Bulgaria; grozdanov@microbio.bas.bg (P.G.); inikolova@microbio.bas.bg (I.N.); evaston@yahoo.com (E.V.-T.); 5 Faculty of Medicine, Sofia University “St. Kl. Ohridski”, 1407 Sofia, Bulgaria; i.grabchev@chem.uni-sofia.bg

**Keywords:** antimicrobial peptides, hemorphins, cotton fabric, absorption, emission, antiviral activity

## Abstract

Some new N- and C-modified biomolecular peptide analogues of both VV-hemorphin-5 and VV-hemorphin-7 with varied amino acids (Cys, Glu, His), 1-adamantanecarboxylic acid, and niacin (nicotinic acid) were synthesized by solid-phase peptide synthesis—Fmoc (9-fluorenylmethoxy-carbonyl) chemistry and were characterized in water solutions with different pH using spectroscopic and electrochemical techniques. Basic physicochemical properties related to the elucidation of the peptide structure at physiological pH have been also studied. The results showed that the interaction of peptide compounds with light and electricity preserves the structural and conformational integrity of the compounds in the solutions. Moreover, textile cotton fibers were modified with the new compounds and the binding of the peptides to the surface of the material was proved by FTIR and SEM analysis. Washing the material with an alkaline soap solution did not show a violation of the modified structure of the cotton. Antiviral activity against the human respiratory syncytial virus (HRSV-S2) and human adenovirus serotype 5 (HAdV-5), the antimicrobial activity against *B. cereus* and *P. aeruginosa* used as model bacterial strains and cytotoxic effect of the peptide derivatives and modified cotton textile material has been evaluated. Antimicrobial tests showed promising activity of the newly synthesized compounds against the used Gram-positive and Gram-negative bacteria. The compounds C-V, H-V, AC-V, and AH-V were found slightly more active than NH7C and NCH7. The activity has been retained after the deposition of the compounds on cotton fibers.

## 1. Introduction

In recent years, more and more attention is paid to antimicrobial peptides in the fight against various pathogenic microorganisms: viruses, bacteria, fungi, and others. In 2020, the COVID-19 pandemic broke out, causing a total collapse in all areas of life. To cope with this pandemic, scientists around the world have begun to develop and test a huge number of compounds, both proven antiviral agents and new as yet undiscovered and potential antivirals. The world’s attention is focused not only on the new vaccines and drugs creation but also on the textile materials for the prevention and control of the invisible enemy, pathogens. The antimicrobial peptides are excellent candidate therapeutic agents that offer high selectivity and specificity, low levels of side effects, and toxicity. The peptides possess distinctive structures and functions by employing sophisticated mechanisms of action [1,2,3]. The great advantage of peptides against viruses consists in the reduced possibility of developing resistance during the treatment [4,5]. Peptides are expensive molecules, but they have some advantages over other kinds of molecules: they are made of natural moieties (α-amino acids), they are biodegradable, and they have negligible toxicity when exploited for external uses. Antimicrobial peptides efficiently inhibit many pathogens, including Gram-negative and Gram-positive bacteria and fungi. Additionally, some antimicrobial peptides have been shown to have anticancer or antiviral activity, such as indolicidin which has activity toward HIV [6]. Peptides can be relatively easily synthesized and modified by using standard synthetic protocols and solid-phase peptide (SPPS) methods. Most of the peptides contain a significant number of precisely located functional groups in their amino acid residues, which possess high-affinity and specific interactions with a target receptor, and they can play a role as ligands for them [7,8]. Peptide scaffolds allow the introduction of desired functional groups in their structure in order to increase biological activity or enhance other wished properties such as easier coating to textiles or other materials, for example, which may increase the possibilities for their application in biochemistry, medicine, and materials chemistry [9,10]. The presence of amino acid residues that have both aromatic moiety and hydrophobic nature, such as Trp, Phe, Tyr, favor the protein binding. According to the literature [11], the presence of free functional (-OH, -SH, -COOH, guanidine, etc.,) groups in the peptide molecules creates a more favorable environment for binding the compound to the cellulosic fibers of textile materials.

For the purposes of our investigations, we preferred the endogenous biopeptides, the hemorphins because in recent years they have been shown a variety of biological activities, such as high affinity to µ-, δ-, and k-opioid receptors [12,13], angiotensin (Ang) IV (AT4) receptor, bombesin subtype 3 receptor (hBRS-3) [14], or corticotropin releasing factor (CRF) receptors [15], etc. Nowadays, hemorphins find their application as therapeutic agents or as biomarkers in various fields of pharmacology (pain, schizophrenia, depression, and addiction treatment) and biomaterial sciences (biomarkers of various disorders e.g., vascular and renal disease, cancer, inflammation), and also, their biological potential as future medicines [16]. Endogenous hemorphin peptides vary from 4 to 10 amino acids and belong to the family of atypical opioid peptides, released during the sequential cleavage of hemoglobin proteins [17,18,19]. Recently, several new analogues of hemorphin-4, hemorphin-5, and hemorphin-7 were synthesized and characterized by our group [20,21,22,23,24]. We demonstrated that not only the position of modification but also the nature of the conjugated group, lead to significant changes in the peptide activity and affinity [20,21,22,23,24,25,26]. Hemorphins bind with high affinity to angiotensin-converting enzyme 2 (ACE2) [27], which is expressed in nearly all human organs in varying degrees. Moreover, both SARS-CoV-2 and SARS-CoV enter host cells via the same ACE2 receptor [28], so targeting this receptor of host cells can block the entry of the virus into the cell, thereby protecting the host from viral infection and pandemic disease COVID-19 [29]. Some microorganisms, especially drug-resistant nosocomial microbes, constantly spread infectious diseases that are transmitted through medical textiles, medical equipment and devices, clothing, gloves, etc. Antimicrobial textiles can prevent the growth of pathogens as well as prevent transmission and spreading of pathogenic microbes or infections from the healthcare environment to the human body [30]. The application of biopeptides in textiles for medical environments has gained attention worldwide since these materials could be useful in the fight against healthcare-associated infections [31,32]. Cotton has widespread use in textiles and in health care environments. Peptides can simply adsorb on cotton through electrostatic interactions, but without covalent bonding they are easily lost. The suitable functionalization of cotton can impart suitable groups for covalent bonding of various polymers and ensure the durability of modification during use [33].

Despite all of the above mentioned, there are no data in the literature about the antimicrobial hemorphin peptide analogues. In the present study, we report on the synthesis and characterization of a series of new N- and C-modified analogues of both VV-hemorphin-5 and VV-hemorphin-7 with varied amino acids (Cys, Glu, His), 1-adamantanecarboxylic acid, and niacin (nicotinic acid) to optimize the physicochemical properties and to enhance their antimicrobial potency. We have also studied the modification of the functionalized cotton fabric with the new antimicrobial peptide compounds. The potential antiviral and virucidal activities of both peptides and textiles material also have been studied.

## 2. Results and Discussion 

### 2.1. Chemistry

**Figure 1:**
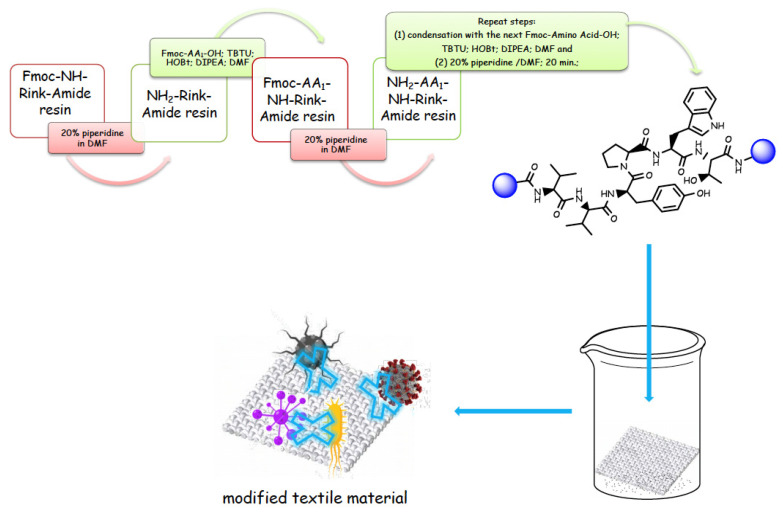
Schematic representation of the solid phase synthesis of the new antimicrobial peptides and their textile application.

We have recently reported structural-textile application and potential antiviral and virucidal activities of both peptides and textiles material of the novel rhodamine B-conjugated hemorphin-4 analogues [34]. Herein, we synthesize, characterize, and investigate the physicochemical properties and antimicrobial activity of both a series of new N- and C-modified analogues of VV-hemorphin-5 and VV-hemorphin-7 and functionalized cotton fabrics (Figure 1). These N- and C-modified peptide analogues, contain different proteinogenic amino acid residues (Cys, Glu, His), 1-adamantanecarboxylic acid, and niacin (nicotinic acid) into the natural endogenous VV-hemorphin-5 and VV-hemorphin-7 molecules. The aim of the present study was to optimize the physicochemical properties and enhance their antimicrobial potency, and also investigate their structural-related properties and a potential textile application using different methods. We have also explored an approach to the structural features by investigating the electrochemical behavior of these peptides, and the potential antimicrobial activities of both the new peptide molecules and modification of functionalized cotton fabrics with these bioactive compounds. The new synthetic hemorphin peptides were prepared manually by solid-phase peptide synthesis (SPPS)-Fmoc (9-fluorenylmethoxy-carbonyl) chemistry using TBTU (2-(1*H*-benzo-triazole-1-yl)-1,1,3,3-tetramethyluroniumtetrafluoro-borate), an efficient peptide coupling reagent. The schematic route of the SPPS and their textile application was summarized in Figure 1. We also used HOBt (hydroxybenzotriazole) as coupling reagents, and DIPEA (*N*,*N*-diisopropylethylamine) as an organic base were added to the reaction media in each step. The chemical structures and molecular formulas, electrospray ionization mass spectrometry data, retention times, and optical rotations of the newly synthesized antimicrobial peptides are presented in Table 1, as different types of modification are given in color.

We synthesized the compounds C-V (Cys-Val-Val-Tyr-Pro-Trp-Thr-Glu), H-V (His-Val-Val-Tyr-Pro-Trp-Thr-Glu), AC-V (Adam-Cys-Val-Val-Tyr-Pro-Trp-Thr-Glu), and AH-V (Adam-His-Val-Val-Tyr-Pro-Trp-Thr-Glu) by replacement of the glutamine (Gln) at the C-terminus with glutamic acid (Glu), first to estimate the influence of Gln on the antimicrobial activity of the peptides, and second the binding of the peptide molecules to the textile material. Aiming to additionally prove the importance of adamantane group and antiviral potency, we introduced the 1-adamantanecarboxylic acid to the N-side, and also amino acids Cys and His of VV-hemorphin-5 to obtain the peptides C-V, H-V, AC-V, and AH-V. Adamantane-containing compounds have long been proven antiviral agents which are currently used in clinical practice: amantadine, rimantadine, and tromantadine [35,36,37]. In their attempts to find an effective way to fight the pandemic situation of COVID-19, scientists are currently synthesizing new biomolecules, as the numerous compounds containing adamantane are constantly increasing. The type and importance of the amino acids that build the peptide chains are of significant importance for their biological activity. The structure–activity relationship has recently been studied, and it has been shown that the peptides containing aromatic (Tyr and Phe), non-polar (Pro and Leu), polar (Gln), and the special case of amino acid Cys residues are extremely important as they act as selective peptide inhibitors targeting the peak SARS-CoV-2 protein [38]. Moreover, we also synthesized new N- and C-modified analogues of VV-hemorphin-7: the compounds NH7C (Nic-Leu-Val-Val-Tyr-Pro-Trp-Thr-Glu-Arg-Phe-Cys) and NCH7 (Nic-Cys-Leu-Val-Val-Tyr-Pro-Trp-Thr-Glu-Arg-Phe) by introducing nicotinic acid (NH7C) and Cys (NCH7) to the N-side, and replacement of Gln with Glu to the C-side of the peptide molecules (NH7C and NCH7). We also introduced Cys to the C-terminus of the peptide NH7C with the intent to evidence the significance of these amino acid modifications, and the amino acids with free functional groups such as -COOH, -SH, guanidine, pyridyl, etc., increased the binding of the peptides with the textiles materials [39,40]. There are data about heterocyclic compounds that act by inhibition of the ion channels of viruses named viroporins, and exert their antiviral activity, for example: pyridine, pyrimidine, histidine, and other derivatives [41]. The peptides typically containing the specific amino acids including arginine, proline, tryptophan, cysteine, and histidine, are shown antimicrobial activities. Their structures are stabilized mostly by hydrogen bonds and van der Waals force of interacting with the membrane lipids. Moreover, the cysteine-rich peptides and their disulfide bond formation are essential for the structural stabilization and biological functions of these peptides [1,2,3]. The bactericidal activity of peptides depends on the positive charge and hydrophobic amino acids that cause bacterial membrane destruction by interacting with phosphatidyl chains [42].

The treatment of the amino acidic scaffold can be attituded as a potentially powerful tool in both bioorganic and medicinal chemistry studies and the evolution of new drugs and materials [43].

### 2.2. Cotton Fabric Modification with Peptides

Traditionally, the even modification of textile materials is achieved by processing in an aqueous solution with various auxiliaries. Recently, we have modified cotton fabric with peptides analogues of hemorphin-4 [34]. The proposed method has achieved very good substantivity and stability of the processing. First, the cellulose macromolecules were functionalized with chloroacetyl chloride. Then, each peptide was dissolved in DMF-water solution, and the fabric was impregnated with the obtained solution. 

Another approach was chosen in this study. The peptides were dissolved in ethanol and applied directly to unmodified cotton fabric.

Thus, several effects were accomplished. First, textile processing was facilitated. Next, the amount of used water was reduced, and in addition, the ethanol, as a solvent, evaporates easy and is a cost-effective environmental alternative to toxic non-degradable auxiliaries.

### 2.3. Physicochemical Characterizations

#### 2.3.1. Spectral Characterizations

**Figure 2:**
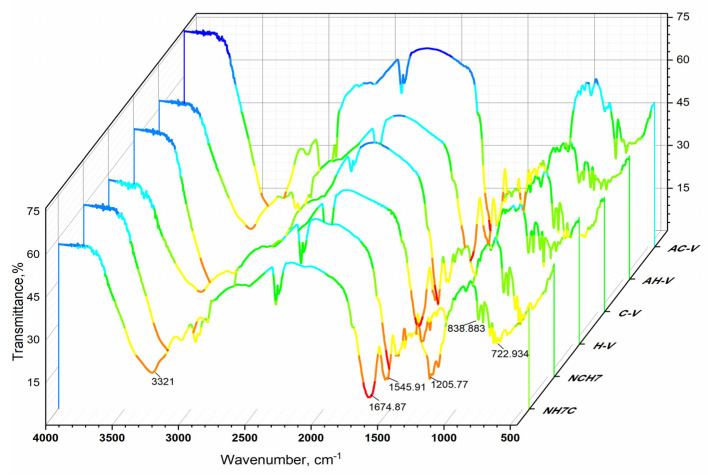
IR-spectra of the peptide derivatives.

The structure characterizations of the test compounds were studied using spectral methods such as UV, IR, and fluorescence analysis. For peptide compounds, IR spectroscopy can be used to tentatively determine the conformational structure of compounds. The main types of bands for this class of compounds are sought, namely: Amide A, 1, 2, and 3. As can be seen, the absorption lines in addition prove the functional groups belonging to the structure of the compounds (Figure 2). In the electromagnetic spectrum of IR spectrometry, a number of characteristic bands corresponding to the amide lines typical of peptide compounds are recorded. The IR spectra of the studied compounds are characterized by amide A bands that are monitored in the 3300–3329 cm^−1^ regions and are due to N-H valence oscillations (ν_NH_) (Figure 2). An assumption for a β-structure can be given by the presence of an intense band located at 1674 nm corresponding to Amide I due to the availability of ν_C=O_ groups. The high absorption maximum at 1530–1545 cm^−1^ (δ_NH_) suggests a β-turn or β-sheet structure [44]. The region of the Amide III band is recognized at 1205–1230 cm^−1^ and is used to confirm β-sheet conformations. In addition to the information from the IR spectra, the presence of certain types of amino acids in the peptide sequence could also lead us to determine the presumed conformational state of the studied peptides. Aromatic amino acids such as Phe, Tyr, and Trp containing (phenyl/indole) rings also prevent the spiral folding of the peptide and therefore the existence of the α-helix. On the other hand, the amino acid Pro is often found in β-folded sheets because it contains a tetrahydropyrrole ring and is not compatible with helix formation [45]. Using literature data on the role of IR spectroscopy in recognizing the type of conformational state of peptide compounds, we can conclude that the probabilistic conformational form of the studied peptides is a beta structure.

**Figure 3:**
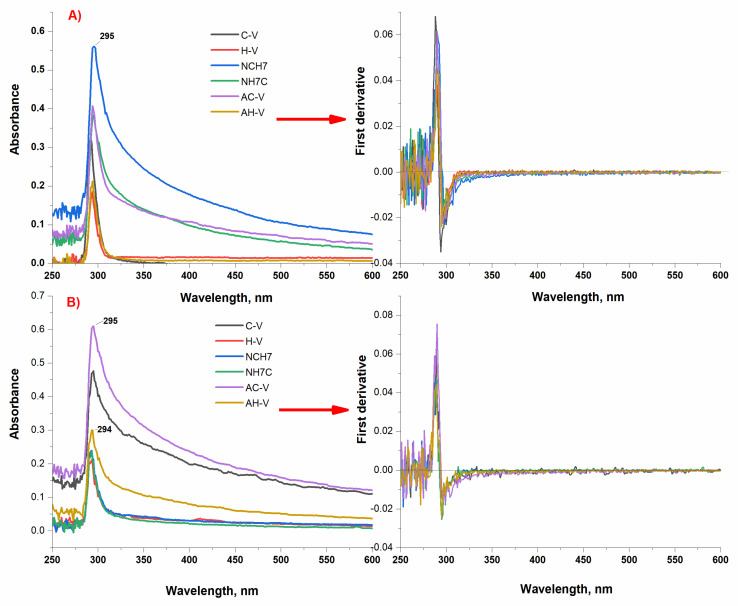
Zero order and first derivative UV-Vis spectrum of peptide derivatives at (**A**) pH 7 and (**B**) pH 9 at follow concentration of the compounds: 1.47 × 10^−4^ mol L^−1^ C-V; 9.80 × 10^−5^ mol L^−1^ AC-V; 8.90 × 10^−5^ mol L^−1^ NH7C; 8.81 × 10^−5^ mol L^−1^ AH-V; 1.02 × 10^−4^ mol L^−1^ H-V; 8.74 × 10^−4^ mol L^−1^ NCH7. Absorbance of all solutions were measured against d·H_2_O.

**Figure 4:**
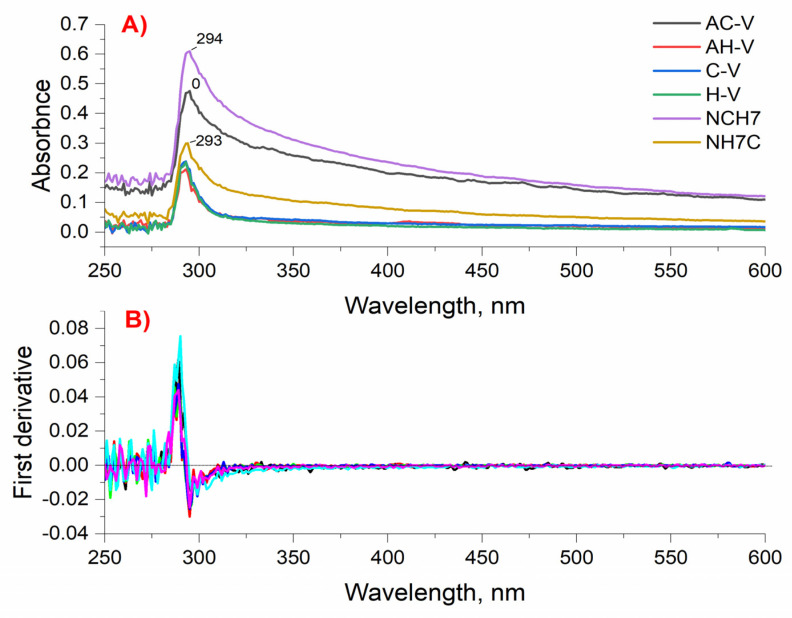
Zero order (**A**) and first derivative (**B**) UV-Vis spectrum of peptide derivatives at pH 3 and follow concentration of the compounds: 1.47 × 10^−4^ mol L^−1^ C-V; 9.80 × 10^−5^ mol L^−1^ AC-V; 8.90 × 10^−5^ mol L^−1^ NH7C; 8.81 × 10^−5^ mol L^−1^ AH-V; 1.02 × 10^−4^ mol L^−1^ H-V; 8.74 × 10^−4^ mol L^−1^ NCH7. Absorbance of the solution was measured against d·H_2_O.

The absorption spectra of the newly synthesized peptide derivatives were obtained in solutions of different pH with micromolar concentrations and showed the typical bands of the absorbing chromophore groups of the amino acid residues of tyrosine and tryptophan. The obtained characteristic bands in the UV region at ≈290 nm for all peptide analogues are mainly due to the π-π* transitions of the phenyl rings of Tyr and Trp [46,47]. Figure 3 shows both the zero-absorption spectrum and the differentiated absorption values. Studies in acidic, neutral, and alkaline media showed similar spectral characteristics of the absorption spectra. There is a difference in the intensity of the analytical signal despite the same concentration of the compound’s solutions and number of peptide bonds. The absorption maxima for the characteristic bands of the compounds in neutral medium are in the near UV range (λmax = 294–295 nm) and are characterized by a single, symmetrical peak for compounds C-V, H-V, and AH-V. For compounds AC-V, NH7C, and NCH7 a wider, asymmetrical peak is observed, which can be attributed to different solubilities of compounds under the specified conditions. The differentiated spectrum crosses the abscissa at 285 nm, which confirms the absorption of certain types of groups. The optical densities at the absorption band maxima show a linear dependence on the concentration and in accordance with the Beer’s law. The absorption maxima in the ultraviolet region at pH 9 and pH 3 are also in the range λmax = 294–295 nm (Figure 3 and Figure 4). However, the intensity of the stripes changes for peptides containing adamantane in their molecule. The absorption activity of the longest chain peptides in alkaline medium decreases, and for the adamantane derivative AC-V a hyperchromic effect is observed, as the width of the right half of the peak increases. Similar behavior is observed in acidic environments. All studied photophysical parameters of the new compounds are summarized in Table 2. The studied peptide compounds showed absorption spectra with high molar absorbency [ε > 1 × 10^5^ L/(mol·cm)] determined at λmax = 294 nm at pH 7 which allows their easy detection at low concentrations.

**Figure 5:**
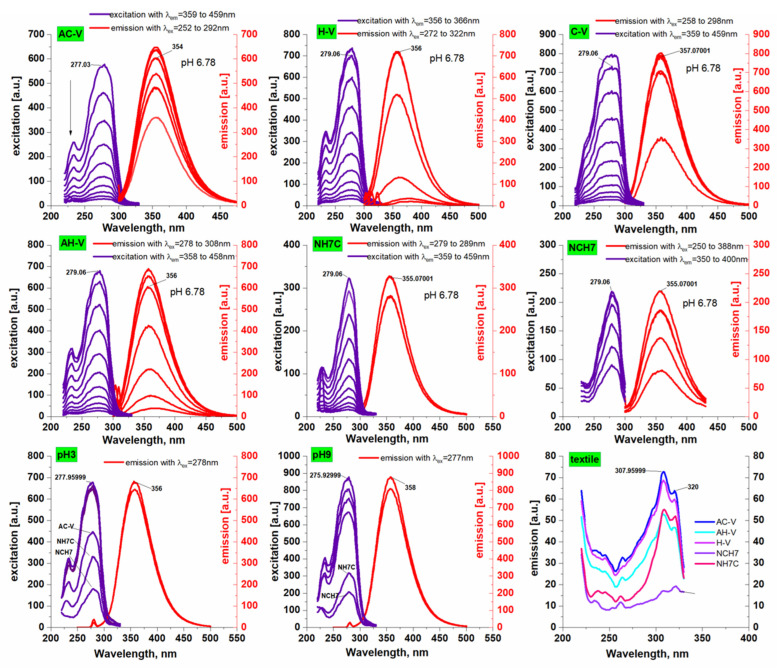
Excitation/emission spectra of investigated peptide derivatives at different pH values and emission spectra of textile-peptide materials.

The fluorescent properties of tyrosine, tryptophan, and phenylalanine-containing peptide derivatives were also studied in the different pH of the chemical media (Figure 5). The main fluorescent characteristics of the peptides are summarized in Table 2. As can be seen, the obtained fluorescent spectra have emission maxima that are not affected by the excitation wavelength in the range λex = 250 to 388 nm. Intense fluorescence spectra show a monomeric state of the molecule without structure loss. The excitation spectra obtained with the peptide compounds are comparable to the absorption ones and it can be concluded that under the excited state the molecules retain their conformational structure. On the other hand, the obtained fluorescence spectra could be evidence of the purity of the test compounds. In investigated solutions, the peptide derivatives show a typical for the fluorophore groups of Trp and Tyr bright fluorescence emission with maximal intensity in the range 354–358 nm depending on the solvent pH that affects the fluorophore charge transfer efficiency. The quantum yields of fluorescence (ΦF) of peptides were calculated using Trp as a standard (ΦF = 0.13 in water) and the obtained values 0.12–0.67 are close to that given in the literature for tryptophan-containing peptides [48].

The values of the quantum yield of compounds are presented in Table 2. The low values of the fluorescent quantum yields, especially for compounds NH7C and NCH7 may be due to physical quenching by volume groups of substituents or due to transition to triplet excited state of the molecule by nonradiative transition or internal singlet–singlet conversion or triplet–singlet, as a result of which heat is released. The low fluorescence quantum yield of this group of compounds could be also explained by the quenching effect of the -COOH groups on the fluorescence of the molecules containing them. As a result, the process of intramolecular photoinduced electron transfer is more likely to occur, resulting in lower fluorescence yields. Moreover, it has been reported that the transfer of an electron from Trp to the carbonyl groups of neighboring peptide bonds is the most important mechanism for intramolecular quenching of fluorescence of Trp from peptides molecules [49]. The Stokes shift as an important feature that shows the differences between the structure of the fluorophore in the ground S0 state and in the first excited state S1 was also calculated for the peptide derivatives (Table 2). The obtained values of the test compounds are in the range from 5650 (AC-V) to 5887 (C-V) cm^−1^. 

Textiles with peptide deposition also showed satisfactory fluorescence. The registered emission maxima at wavelengths as in the model solutions are indicative of maintaining the integrity of the molecule in the chemical treatment of the material. However, asymmetry of the peaks is observed due to the processes of aggregation of the molecules, which are also proved by the SEM analyzes. The decrease in the intensity of the emission maximum may be due to the presence of bulky glucoside monomers of the cellulose fibers to which the peptide derivatives bind, probably by forming hydrogen bonds. It has been shown in the literature that bulky residues can cause fluorescence quenching in a number of compounds. Moreover, the emission wavelength appears at 307 and 320 nm. By themselves, the two fluorophores that are mainly responsible for the fluorescence of the peptide molecule are tryptophan and tyrosine. The molecule of tryptophan and tyrosine emits radiation at 302 nm for tyrosine and 355 nm for tryptophan, respectively, at an excitation wavelength of 278 nm. In the textile material, both characteristic peaks for these chromophores appear with tryptophan blue shift of the maximum, probably due to the organic proton medium in which the peptides are prepared for the treatment of the material as it is known that the indole is highly dependent upon polarity and/or local environment [50].

#### 2.3.2. Electrochemical Characterizations

**Figure 6:**
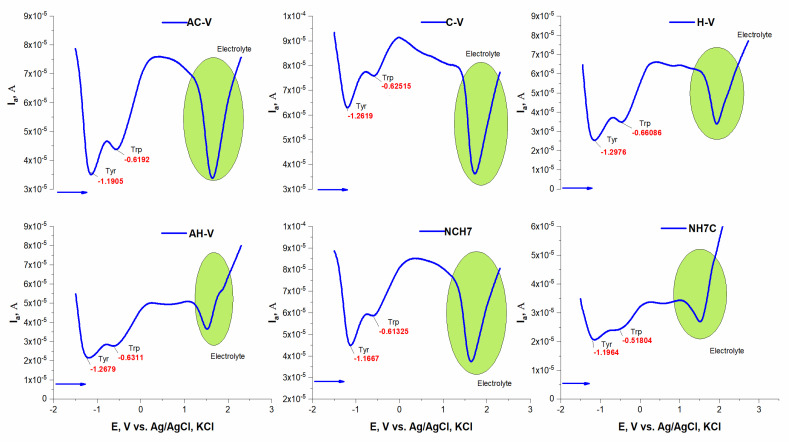
Plots of Ep, [V] vs. I, [A] in differential pulse voltammetric determinations of the peptide derivatives at pH 6.87.

**Figure 7:**
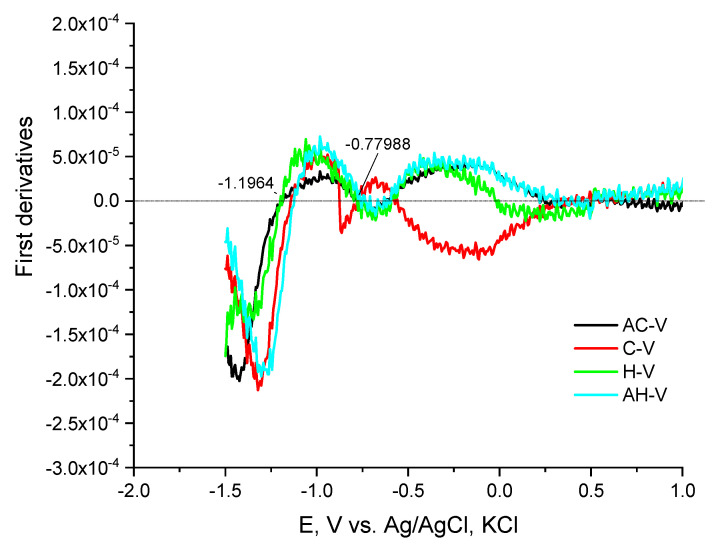
Plots of Ep, [V] vs. first current derivatives of differential pulse voltammetric determinations of the peptide derivatives.

**Figure 8:**
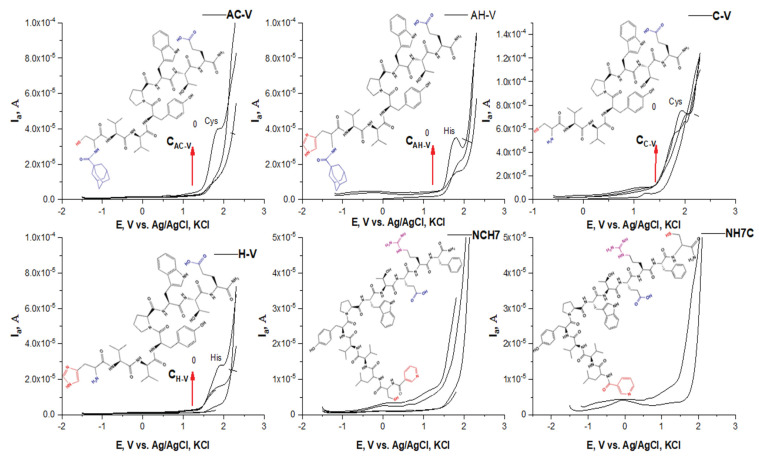
Plots of Ep, [V] vs. I, [A] in differential pulse voltammetric determinations of the peptide derivatives at pH 7.34.

The electrochemical behavior of peptide compounds was also studied to evaluate their interaction with solid surfaces (e.g., the surface of a glass carbon electrode). The peptide compounds included as antimicrobial agents in textile materials are present in micro quantities, therefore it is necessary to apply hypersensitive methods for their detection and monitoring of their structural performance. The interaction of peptide derivatives with hard surfaces (e.g., electrode surface) is not only a fundamental phenomenon but also a key to several important and new applications of these compounds as biosensors for the detection of antimicrobial biomaterials. The peptide molecule is often complex in nature, with differences in characteristics such as solubility and charge, highly dependent on both naturally occurring amino acids and the exact amino acid sequence [51]. The main transfer of a compound to the solid surface of the electrode is by adsorption and diffusion, and it is often reported that hydrophobic surfaces adsorb more compounds with large molecules than hydrophilic ones [52,53]. The conformational state of the peptide molecule also plays a role in the electronic exchange between the electrode and the electroactive centers of the peptides, and usually, the inhibition of the electroactive groups reduces the possibility of transfer and blocks the electrochemical activity of the molecule. Such an orientation can also increase the distance between electroactive amino acids and electrodes and thus also increase the difficulty of electron transfer [54]. Direct electronic transfer of peptides and proteins has been reported to mainly involve compounds containing electroactive groups of amino acids such as tyrosine [55,56], tryptophan [57], and histidine [58], cysteine and methionine [55], oxidizable at carbon electrodes [59]. The oxidation process of tyrosine is associated with the formation of a thermodynamically unstable radical from the hydroxyl group attached to the benzene nucleus of the peptide structure. The desire to obtain a more stable radical structure leads to the formation of electroactive orthoquinone reducing reversibly to catechol by a two-electron-proton mechanism [53]. The oxidation of tryptophan takes place in two pH-dependent irreversible oxidation steps, as follows: (1) first oxidize the pyrrole ring and (2) indole electrochemical hydroxylation of the benzene moiety to form two electroactive products adsorbing on the electrode surface [56]. For the amino acid cysteine, three successive oxidation reactions on the GCE surface were observed involving the oxidation of the sulfhydryl group and the formation of a radical undergoing nucleophilic attack from water as a solvent to give an intermediate that oxidizes in the second step to cystic acid. The voltammetric behavior of the peptide compounds was studied in phosphatic buffer solution at pH 6.87 and 7.34 by differential pulse voltammetry. In a slightly acid medium, the production of an anode peak at the potential is proved −1.19 V by shifting to higher potentials, both with an increasing number of amino acid residues in the peptide molecule and with increasing pH of the medium. This could be associated with improved solubility, especially when the molecule is larger. What is striking is that the analytical signals characteristic of electroactive amino acids are detected, but significantly shifted at lower potential values despite their anodic oxidation (Figure 6). The peaks corresponding to the oxidation of tyrosine and tryptophan interact and partially overlap (Figure 6). By constructing the graphical dependence of the first derivative of the current data on the occurrence potential of the signals, a clear distinction and separation of the two peaks is achieved and the place of their occurrence is proved (Figure 7). However, it was found that at potentials around −1.19 the tyrosine part is oxidized, and at −0.600 the indole part of tryptophan (Figure 6 and Figure 7). It is felt that both the conformational state of the molecule and the functional groups of adjacent amino acid residues close to the electroactive groups affect the electron exchange and the degree of oxidation. At higher pH values, the appearance of both histidine and cysteine peaks in the compounds containing it is also observed (Figure 8). Moreover, up to a certain increase in the analyte concentration, a proportional increase in the intensity of the peak is observed, without shifting the place where it appears. This indicates a diffusion-controlled process with subsequent saturation of the electrode surface. It is noticed that in the case of long-chain peptides (NH7C and NCH7) the signals of the cysteine part are not registered, most likely due to the presence of spatial obstruction. The half-widths of the tyrosine and tryptophan peaks correspond to a quasireversible process with one proton and one electron exchange process with the electrode space.

#### 2.3.3. FTIR Analysis of Textile Materials

**Figure 9:**
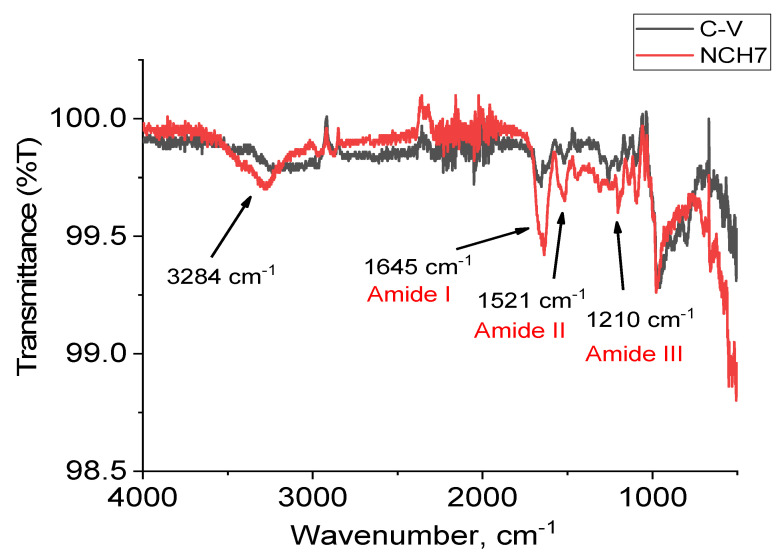
Subtracting FTIR spectrum of cotton fabric from the spectra of cotton fabric modified with C-V and NCH7.

The study of the interaction between each peptide and cotton fabric has been performed by FTIR spectroscopy. The cellulose macromolecules can form hydrogen bonds with amide groups of peptides. As the quantity of peptides used for the cotton modification is small, the more informative are the obtained spectra by subtracting a reference spectrum of pure cotton from a spectrum of cotton fabric modified with each peptide. Figure 9 shows as an example the subtracting spectrum of the fabric sample modified with peptide C-V compared with cotton fabric modified with NCH7, as an example. In the subtracting spectrum of peptide NCH7, the band, due to N-H valence oscillations (ν_NH_), appears with a maximum at 3284 cm^−1^ with small intensity, while in peptide C-V, this band is missing. The peptide NCH7 has a longer chain than peptide C-V and is probably part of amide groups not involved in hydrogen bond formation. Bands in the regions typical for Amide I, Amide II, and Amide III are also less intensive for peptide C-V. In peptide NCH7, these bands are more intensive because of free amide bonds, uninvolved in hydrogen bond formation. The spectra of the other samples are presented in the Supplementary Materials.

### 2.4. SEM Analysis of Textile Materials

**Figure 10:**
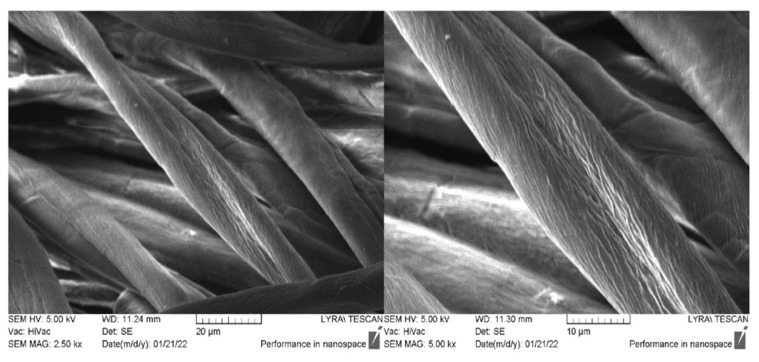
SEM images of the untreated fabric at different magnifications.

**Figure 11:**
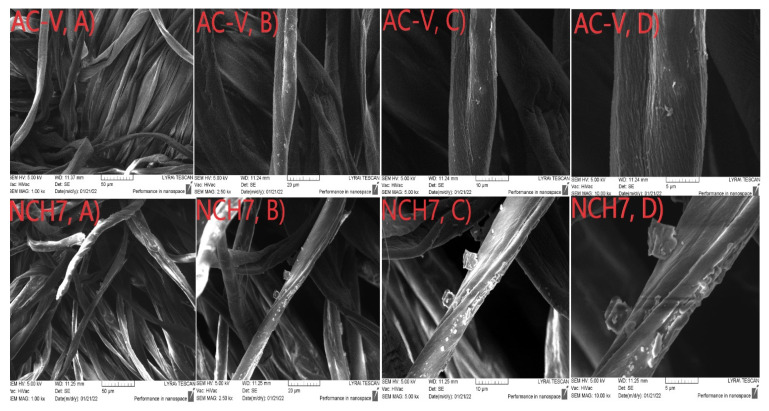
SEM images of cotton fabrics at different magnifications: treated by AC-V (40%) (from (**A**–**D**)): 50–5 µm) and NCH7 (40%) (from (**A**–**D**)): 50–5 µm).

The morphology and the presence of peptide compounds on the surface of the cotton material are also characterized by SEM analysis. Figure 10 shows the micrographs of the untreated cotton fibers at different magnifications. It is well visible that the characteristic cotton fiber shape is of a flat twisted ribbon. The longitudinal arrangement of the cellulose microfibrils to the fiber axis is the reason for its rough surface [60]. After the material processing with new synthesize peptides, the typical structure of the fabrics is preserved, and the individual fibers are well visible. Figure 11 shows the cotton fabric treated with a short-chain peptide (AC-V) and a longer chain peptide (NCH7). Deposition of peptide AC-V on the surface of fibers leads to the formation of an uneven layer containing aggregates with irregular form (Figure 11A–D).

The coating of the fabric with a longer chain peptide leads to forming an uneven layer of aggregates of different sizes and shapes (Figure 11). The structure of the NCH7 peptide and its more difficult dissolution before application to the fabric compared to the AC-V peptide are the probable reasons for the different topology of the aggregates and their distribution and interaction with the fibrous surface.

### 2.5. Fastness Testing

**Figure 12:**
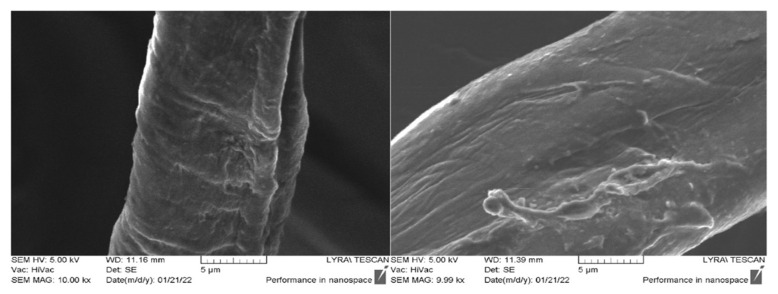
SEM images after washing the material in a soapy alkaline solution with 15 min of soaking with stirring.

An essential requirement for textile materials modification to obtain new properties is their stability in the conditions of their use, for example, as a medical material. Therefore, we investigated the effect of washing on peptide-treated textile materials by the immersion method in alkaline soapy water. Figure 12 shows the SEM micrographs of the fiber surface of sample AC-V after washing. It is visible that more of the aggregates disappear after treatment in an alkaline soap solution for 15 min. However, even after these severe processing conditions of fabric, the peptide layer is visible on the fiber surface.

### 2.6. Virological Activity

Peptides with antiviral activity against influenza can be divided into three main groups: Entry blocker peptides (for example FluPep); peptides that display virucidal activity, disrupting viral envelopes (e.g., Melittin); and a set of peptides that interact with the viral polymerase complex and act as viral replication inhibitors such as PB1-derived peptides [5]. The novel N- and C-modified analogues of VV-hemorphin-5 and VV-hemorphin-7 demonstrated a virucidal effect against the human respiratory syncytial virus (HRSV-S2) for different time intervals (30 and 60 min). The most active is H-V (His-Val-Val-Tyr-Pro-Trp-Thr-Glu) and AC-V (Adam-Cys-Val-Val-Tyr-Pro-Trp-Thr-Glu) peptide analogues, which are analogues of natural VV-hemorphin-5. Both the H-V and AC-V compounds contain glutamic acid (Glu) instead of VV-hemorphin-5 naturally glutamine (Gln) at the C-terminus. Furthermore, the AC-V peptide containing lipophilic adamantyl building block and enigmatic amino acids Cys to the N-side of peptide molecule for the difference from H-V analogue containing heterocyclic basic amino acid His. The difference between other compounds is the varied amino acid residues with free functional groups: -COOH, -SH, guanidine, and pyridyl, and their different polarity, charge, hydrophobicity, etc., that they attach to the ended peptide molecule. Compounds H-V and AC-V showed higher virucidal activity against HRSV-S2 at 60 min, unlike compound NH7C which is more active at 30 min (Table 3). All of the peptides did not show any virucidal activity against HAdV-5 in both 30 and 60 min intervals. Perhaps this is due to the structure of HAdV-5 (non-enveloped virus) which does not have a lipid bilayer envelope thus making them more resistant to chemicals.

For a more in-depth study of the virucidal effect against both HRSV-S2 and HAdV-5, cotton fabrics with VV-hemorphin-5 and VV-hemorphin-7 peptide analogues have been also studied. Unfortunately, the virucidal effect of the textile materials was low, and was only against HRSV-S2 compared to hemorphin peptides (Table 4).

**Figure 13:**
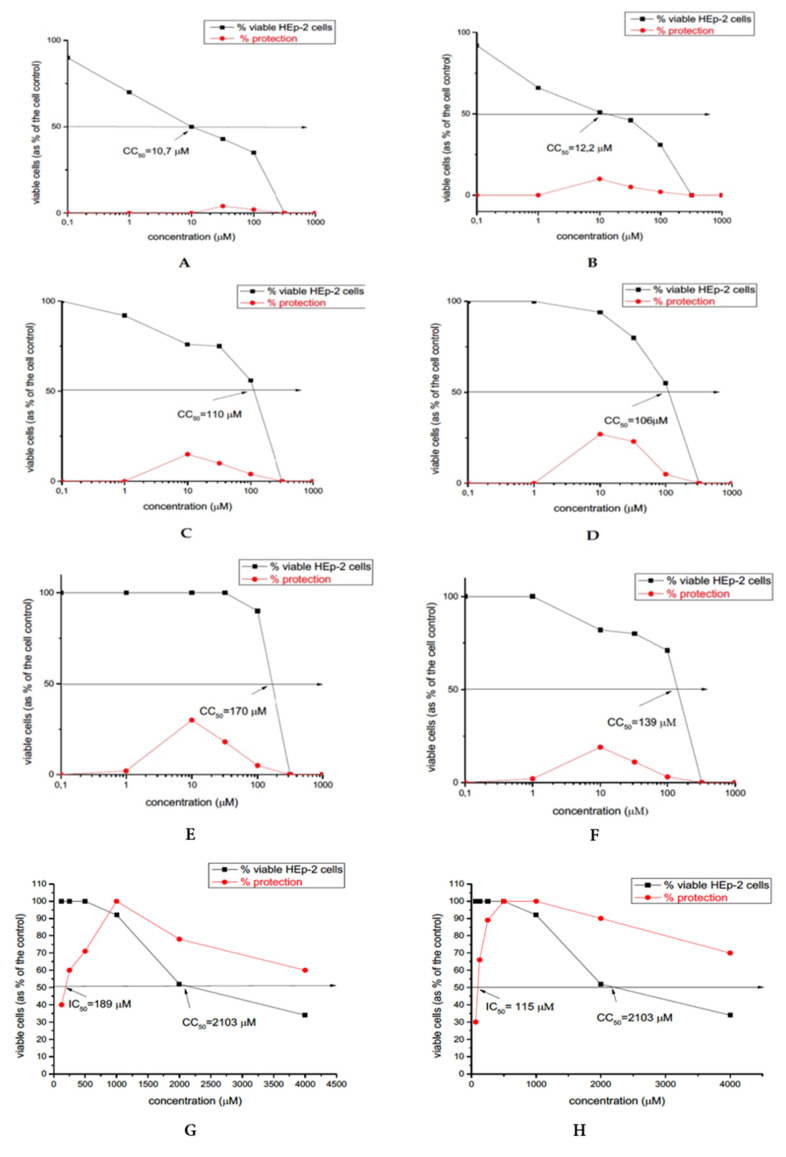
Antiviral activity curve (in red) and cytotoxicity curve (in black) of: (**A**) C-V; (**B**) H-V; (**C**) AC-V; (**D**) AH-V; (**E**) NH7C; (**F**) NCH7; (**G**) cytotoxicity and antiviral activity of ribavirin on the replication of HRSV in HEp-2 cells and (**H**) cytotoxicity and antiviral activity of ribavirin on the replication of HAdV-5 in HEp-2 cells.

The newly obtained antimicrobial compounds shown weak virucidal activity. NH7C and NCH7 are more potent as opposed to the other peptide compounds (see Figure 13). The most potent peptide analogues NH7C and NCH7 contain nicotinic acid, Arg, and Cys in their molecules. Our experimental data suggest that in the new hemorphin peptides containing varied proteinogenic amino acids (Cys, Glu, His), 1-adamantanecarboxylic acid, and niacin residues, not only the position of the modification but also the nature and length of the amino acid sequence lead to significant changes in peptide activity and affinity. The results suggest that the incorporation of different amino acids and pharmacophore residues at the N- and C- terminus both VV-hemorphin-5 and VV-hemorphin-7 scaffolds deserve further evaluation in antiviral and virucidal effects.

We used as reference substance the well-known synthetic drug ribavirin which has been used for several decades against many RNA and DNA viruses. Ribavirin is a synthetic guanosine nucleoside and antiviral agent that interferes with the synthesis of viral mRNA. The cytotoxicity of the studied compounds is higher compared to the cytotoxicity of ribavirin which is a drug designed for intake administration in contrast to the tested compounds, which are designed for processing and immobilization on fabrics. 

The cytotoxicity data of the compounds and referral substances in HEp-2 cell culture are shown in Table 5.

### 2.7. Antibacterial Activity

Initially, the compounds were tested on an agar medium using commercial discs with gentamicin (10 µg) as a standard against *B. cereus* and *P. aeruginosa* used as model bacterial strains. As a result, no inhibition zones were observed around the discs with the compounds against both tested strains. With gentamicin, zones (mm in diameter) of 15 mm against B. cereus, and 12 mm against P. aeruginosa, were measured. This result can be explained by the insolubility of the compounds in water and hence their inability to diffuse into the agar medium and to have direct contact with the bacterial cells.

**Figure 14:**
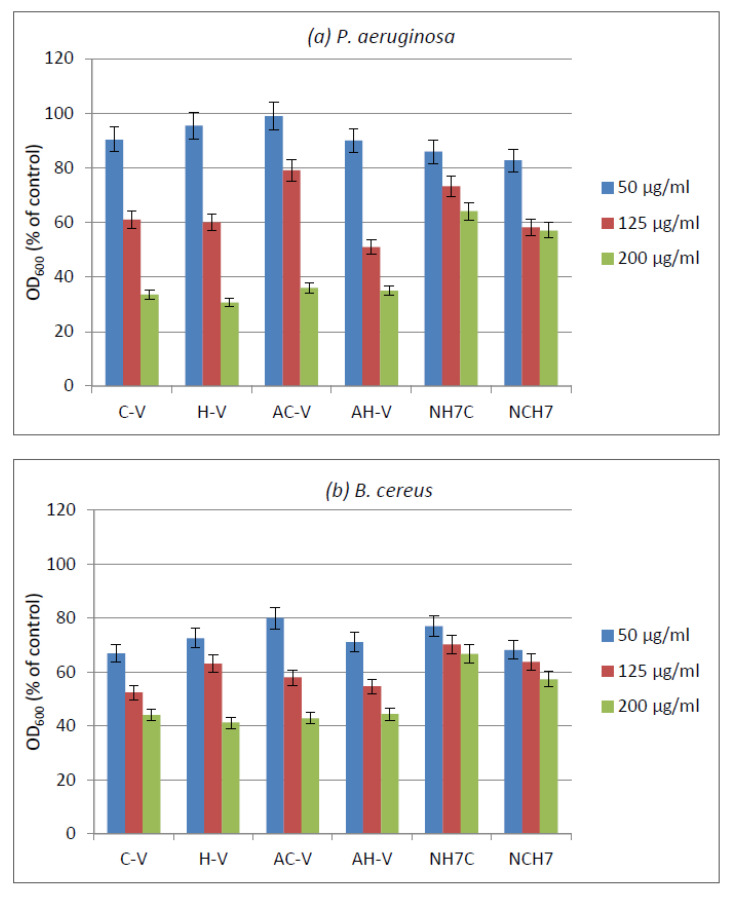
Growth of model bacteria (**a**), *P. aeruginosa* and (**b**), *B. subtilis* at different concentrations in micrograms (µg/mL) of the investigated compounds.

For this reason, the antimicrobial activity of the compounds was evaluated against both bacteria in a liquid medium. The results showed different activities of the compounds depending on their species and type of strains (Figure 14). Gram-positive *B. subtilis* was slightly more sensitive than Gram-negative *P. aeruginosa* which can be explained by the differences in the cell wall structures of Gram-positive and Gram-negative bacterial cells. At a concentration of the compounds 200 µg/mL, the samples C-V, H-V, AC-V, and AH-V have shown similar inhibition of the growth of *P. aeruginosa* (in the range 64–66%) followed by the samples NCH7 and NH7C (43% and 36% growth reduction, respectively) (Figure 14a). Against Gram-positive *B. subtilis*, a similar trend was observed at a concentration of 200 µg/mL with growth inhibition of 56–59% by C-V, H-V, AC-V, and AH-V samples, followed by NCH7 (43%) and NH7C (33%) samples (Figure 14b). At concentration 125 µg/mL, a similar better-pronounced trend was observed at both tested strains.

**Figure 15:**
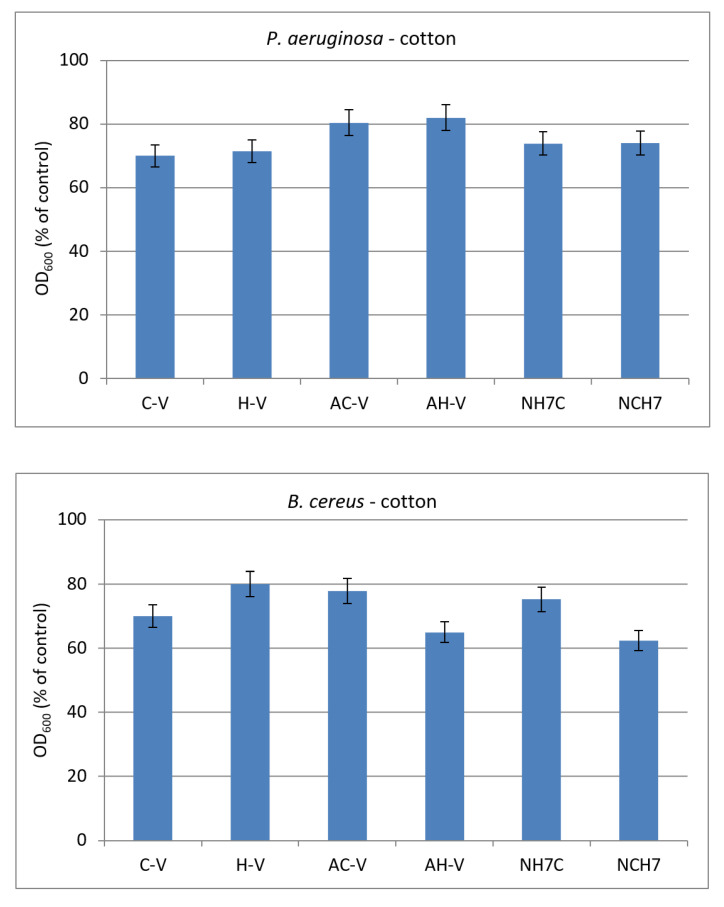
Growth of model strains *P. aeruginosa* and *B. cereus* in presence of cotton fabrics treated with the compounds.

The antimicrobial activity of cotton fabric treated with the compounds was evaluated by the reduction in bacterial growth in MPB. The treated cotton fabrics showed different growth reductions depending on the strain and the type of the compounds (Figure 15).

Antimicrobial tests showed promising activity of the newly synthesized compounds against the used Gram-positive and Gram-negative bacteria. The compounds C-V, H-V, AC-V, and AH-V were found slightly more active than NH7C and NCH7. The activity has been retained after the deposition of the compounds on cotton fabric.

## 3. Materials and Methods

### 3.1. Synthesis of the Peptides (C-V, H-V, AC-V, AH-V, NH7C and NCH7)

All reagents and solvents were analytical or HPLC grade and were bought from Fluka or Sigma-Aldrich, and used without further purification. The protected amino acids and Fmoc -Rink Amide MBHA Resin were purchased from Iris Biotech (Germany). The 3-functional amino acids were embedded as follows: Glu—as Fmoc-Glu(tBu)-OH, Arg—as Fmoc-L-Arg(Pbf)-OH, Tyr—as N^α^-Fmoc-Tyr(tBu)-OH, Thr—as N^α^-Fmoc-Thr(t-Bu)-OH, Trp—as N^α^-Fmoc-Trp(Boc)-OH, His—as Fmoc-L-His(Trt)-OH, and Cys—as Fmoc-L-Cys(Trt)-OH. 

The solid-phase peptide synthesis by Fmoc chemistry was used to obtain new antimicrobial hemorphin analogues. We used Fmoc-Rink-Amide MBHA resin as a solid phase carrier to get on the C-terminal amide analogues and 2-(1*H*-benzotriazole-1-yl)-1,1,3,3-tetramethylaminium tetrafluoroborate (TBTU) was used as a coupling reagent. The coupling reactions were performed using amino acid/TBTU/HOBt/DIPEA/resin with a molar ratio of 3/2.9/3/6/1, in a 1:1 mixture of DMF/DCM. Fmoc group removal is carried out in 20% piperidine solution *N*,*N*-dimethylformamide (DMF) at every step. After each reaction step, the resin was washed with DMF (3 × 1 min), isopropyl alcohol (3 × 1 min), and CH_2_Cl_2_ (3 × 1 min). The coupling and deprotection reactions were checked by the standard Kaiser test [61,62]. The synthesized peptide was removed from the resin using a mixture of 95% trifluoroacetic acid (TFA), 2.5% triisopropylsilane (TIS), and 2.5% water. The peptide was obtained as a filtrate in TFA and thawed with cold dry diethyl ether, after which the resulting precipitate was filtered, and lyophilized after dissolution in water. The final products were obtained in the form of crude peptides, which were dissolved in a mixture of water and acetonitrile until completely dissolved. The peptides were obtained as white powders with a purity of >97% as determined by analytical HPLC. The structures were confirmed by ESI-MS. The purity of the peptides was monitored on a reversed-phase high-performance liquid chromatography (RP-HPLC), column: SymmetryShield^TM^ RP-18, 3.5 μm, (50 × 4.6 mm), flow: 1 mL/min, H_2_O (0.1% TFA)/CH_3_CN (0.1% TFA), gradient 0→100% (45 min) and 100% (5 min). The crude peptides were purified using semi-preparative HPLC, column XBridge^TM^ Prep C18 10 µm (10 × 250 mm), flow: 5 mL/min, H_2_O (0.1% TFA)/CH_3_CN (0.1% TFA), gradient 20→100% (50 min). The newly synthesized analogues of both VV-hemorphin-5 and VV-hemorphin-7 were checked by optical rotations and all of the analytical data are shown in Table 1 and also the Supplementary Materials.

### 3.2. Physicochemical Characterization

#### 3.2.1. Spectral Measurements

##### Apparatus

The UV-Vis spectra were carried out on Varian-Cary with a 1 cm path length synthetic quartz glass cells spectrophotometer. The fluorescence spectra of the peptide derivatives were recorded via a Cary Eclipse, (Agilent, New York, NY, USA) spectrofluorometer in the range 200–900 nm with resolution 0.5 nm and double-grating monochromators in excitation and emission. The IR spectrum was recorded in a potassium bromide (KBr) pellet with a Varian 660 FTIR spectrophotometer, and the spectra was recorded in the range 4000–500 cm^−1^ using Fourier transform infrared spectroscopy (FT-IR). The samples were scanned 256 times at a resolution of 2 cm^−1^ for more precise results. The IR spectrum of the compounds deposited on the textile material were reordered using Agilent Cary 630 FTIR (USA, with Diamond ATR interface module and Agilent MicroLab PC software. High-resolution electrospray mass spectrometry on a Q Exactive mass spectrometer (Thermo Fisher Scientific Inc., Waltham, MA, USA) equipped with TurboFlow TM Transcend chromatography system (Thermo Fisher Scientific Inc., USA) and heated electrospray ionization (HESI II) source was used for confirmation of the molecular mass and purity of the compounds. A computing software (XCalibur^®^ 2.4, Thermo Fisher Scientific Inc., USA) was applied for data acquisition and processing. The apparatus worked at following instrumental parameters: spray voltage—4.0 KV, sheath gas—30 AU, auxiliary gas—12 AU, capillary temperature—300 °C, spare gas—3 AU, heater temperature—300 °C with full scan experiments in a range of 120—2000 *m*/*z* at 140,000 resolutions. A modular circular polarimeter (Anton Paar Opto Tec GmbH, Seelze, Germany) was used in carrying out the optical rotations of the peptides. 

SEM/FIB LYRA I XMU SEM (TESCAN) with the following analytical characteristics electronic source: tungsten heating filament; resolution–3.5 nm at 30 kV, accelerating voltage—200 V to 30 kV; EDX detector: Quantax 200 by BRUKER, spectroscopic resolution at Mn-Ka and 1 kcps 126 eV was used to capture images of textile material to prove the presence of peptide derivatives on textile fabrics.

##### Solutions for UV-Vis and Fluorescence Analysis

Standard solutions of the peptide compounds were prepared by dissolving the dry substance in water:methanol = 1:1 and the concentrations are as follows: 8.82 × 10^−4^ mol L^−1^ C-V; 7.84 × 10^−4^ mol L^−1^ AC-V; 7.12 × 10^−4^ mol L^−1^ NH7C; 7.05 × 10^−4^ mol L^−1^ AH-V; 8.16 × 10^−4^ mol L^−1^ H-V; 6.99 × 10^−4^ mol L^−1^ NCH7. UV-Vis and fluorescence spectra were measured in dilute solutions with concentrations: 1.47 × 10^−4^ mol L^−1^ C-V; 9.80 × 10^−5^ mol L^−1^ AC-V; 8.90 × 10^−5^ mol L^−1^ NH7C; 8.81 × 10^−5^ mol L^−1^ AH-V; 1.02 × 10^−4^ mol L^−1^ H-V; 8.74 × 10^−4^ mol L^−1^ NCH7. A series of solutions with the indicated concentrations were prepared at pH 10 (0.00013 mol L^−1^ NaOH) and pH 3 (0.0005 HCl mol L^−1^).

The quantum yields of fluorescence (ΦF) were determined relatively to tryptophan as a standard (ΦF = 0.13 in water) [48].

#### 3.2.2. Electrochemical Measurements

A Metrohm 797 VA trace analysis apparatus with three-electrode voltammetric cells connected to a 797 VA stand with experimental control and data acquisition was used for the electrochemical measurements. The electrodes in the cell are as follows: glass-carbon (GC) working electrode, platinum wire as auxiliary, and Ag/AgCl, KCl as comparative electrodes. Cyclic and differential pulse voltammetric modes were used to record the analytical signals. Each voltmaperogram was taken after gentle stirring and purging of 6.0 mL electrolyte solution containing aliquots of the analyte (100.0 to 400.0 μL) with pure nitrogen (99.999%) for 300 s. The temperature in the laboratory medium was maintained at 25 °C. 

Two types of solutions were used during the voltammetric analyses: standard aqueous methanolic solutions of the peptide compounds with the concentrations given in Section 3.2.1 (Solutions for UV-Vis and Fluorescence Analysis) and commercial buffer solutions with pH: 6.78.

### 3.3. Cotton Fabric Modification and Characterization

#### 3.3.1. Modification of Cotton Fabric with Peptides

Each peptide (1% of the weight of the cotton fabric) was dissolved in ethanol at room temperature for peptides C-V, H-V, AC-V, and AH-V and at an elevated temperature of 40 °C for peptides NH7C and NCH7. Then the washed and dried fabric sample were impregnated with the corresponding peptide solution at a liquor-to-good ratio of 2:1 and dried in the open air. The resulting textile samples were dipped in deionized water for 2 h and dried again at room temperature.

#### 3.3.2. Fastness Testing

The cotton fabric treated with peptide compounds was tested for the fastness of the peptides on the material when washed with water and soapy water solution [63]. The stability of the modified cotton material was proven by SEM analysis.

### 3.4. Virology

#### 3.4.1. Cytotoxicity Assay 

Different concentrations of the peptides (with 0.1 mL/well), properly diluted in a maintain medium were used for the inoculation of monolayer cells in 96-well plates (Costar^®^, Corning Inc., Kennebunk, ME, USA). A humidified atmosphere at 37 °C and 5% CO_2_ for 48 h was used for the incubation of the cells. The following procedures were used after a microscopic evaluation: (1) The maintenance medium containing the test compound was removed and the cells were washed; (2) 0.1 mL of maintenance medium supplemented with 0.005% neutral red dye was added to each well; (3) the cells were incubated for a long time (3 h) at 37 °C. When incubation finished, the neutral red dye was removed, then the cells were washed once with PBS, and 0.15 mL/well desorb solution (1% glacial acetic acid and 49% ethanol in distilled water) was added. The optical density (OD) of each well was read at 540 nm using a microplate reader (Biotek Organon, West Chester, PA, USA). The material concentration that reduced the cell viability by 50% when compared to the untreated control is defined as 50% cytotoxic concentration (CC50). 

#### 3.4.2. Antiviral Activity Assay 

For checking the viral load, the antiviral screening based on the viral yield reduction technique was used. The cytopathic inhibition assay (CPE) utilizes 100 CCID50 loading in 0.1 mL of a monolayer of confluent cells in 96-well plates. After 1 h of virus adsorption, the extract was added in various concentrations, and cells were incubated for 48 h at 37 °C. The viable cells were stained according to the neutral red uptake procedure and the percentage of CPE inhibition for each concentration of the test sample was calculated using the following formula: % CPE = [ODtest sample − ODvirus control]/[ODtoxicity control − ODvirus control] × 100. The concentration of the material inhibition 50% of viral replication when compared to the virus control was defined as the 50% inhibitory concentration (IC50). The ratio CC50/IC50 was calculated to evaluate the selectivity index (SI).

#### 3.4.3. Virucidal Assay 

The investigated peptide analogues (C-V, H-V, AC-V, AH-V, NH7C, and NCH7; solutions with 1 mL) were tested for antiviral activity against the human respiratory syncytial virus (HRSV-S2) (105.3 CCID50) or respectively human adenovirus type 5 (HAdV5) (106.3 CCID50) following the testing procedure given in [38]. The cotton fabrics modified with these peptides were also tested for antiviral activity as cut identical pieces of the textiles (1 cm^2^) were immersed in a viral suspension (100 µL) for the respective times (30 and 60 min). A control from nonmodified textile was used for the comparative sample.

### 3.5. Antibacterial Activity

#### 3.5.1. Test Microorganisms

The antibacterial activity of the investigated compounds was tested against Gram-positive Bacillus cereus and Gram-negative Pseudomonas aeruginosa used as model strains (Collection of the Institute of Microbiology, Bulgarian Academy of Sciences). Microbial cultures were maintained at 4 °C on meat-peptone agar (MPA) slants and transferred monthly.

#### 3.5.2. In Vitro Antimicrobial Assay

The antibacterial activity of the new compounds against the model strains was tested in meat-peptone broth (MPB). Stock solutions of the investigated compounds (0.5% in DMSO) were prepared and further diluted in test tubes with MPB to final concentrations 50, 125, and 200 µg/mL. Inocula were prepared by diluting the overnight cultures with 0.9% NaCl to a 0.5 McFarland standard. After inoculation with each standardized microbial suspension, the tubes were incubated at the appropriate temperatures for 24 h under shaking (at 240 rpm). Positive controls (compounds and MPB, without inoculum) and negative controls (MPB and inoculum, without compounds) were also prepared. The turbidity of the medium at 600 nm (OD600) was used for the evaluation of the microbial growth. The % microbial growth was determined on the basis of the positive control which was considered as 100%. Three replicates of the experiments to estimate the standard deviation of the series were performed (Sr < 5%).

#### 3.5.3. Antibacterial Activity of Cotton Fabrics Treated with the Compounds

The antibacterial activity of cotton fabrics treated with 1% solutions of the compounds was investigated in MPB against the model B. cereus and P. aeruginosa strains. Test tubes with sterile MPB and square cotton specimens (10 mm × 10 mm) were inoculated with a cell suspension of each bacterial culture. Tubes with untreated cotton samples and without specimens were also prepared as controls. After 24 h incubation under shaking, the specimens were removed and OD600 was determined. The antimicrobial activity of the treated cotton samples was evaluated by the reduction of OD600 after incubation compared to the control sample. All assays were performed in triplicate and the average was taken (Sr < 5%).

## 4. Conclusions

During the last two years, the need to study textile materials as the “first” safeguards in the prevention of living organisms from pathogens has grown significantly. Along with general immunomodulation with an overall effect against viruses and other pathogens, blocking the path of infection through the oral cavity through the use of textile masks would further help prevent touching the face, which is actually the main source of infection, thus the mask plays the role of a barrier to saliva droplets or nasal secretions when sneezing and coughing. In this regard, modifying a textile material with biologically active compounds would strengthen its defense mechanism and improve its application. Our work showed a study on the possibility of modifying cotton material with peptide derivatives containing different amino acid residues. Preliminary studies of their physicochemical properties in model solutions have shown satisfactory results for keeping the structure of the compounds. Fluorometric analyses have shown that in neutral and slightly alkaline aqueous solutions the compounds retain their structural integrity. Data on their relationship to hard surfaces were obtained by electrochemical studies. Differences in the electrochemical oxidation of the peptides at low and high concentrations were observed. Higher concentrations and longer amino acid residues lead to saturation of the solid surface of the electrode, the adsorption process, and no signal. Moreover, a textile cotton material was modified with the new compounds and the binding of the peptides to the surface of the material was proved by FTIR and SEM analysis. Molecule size and amino acid sequence have also been shown to be important factors in determining the biological activity of compounds. Compound H-V showed the best antiviral activity against the human respiratory syncytial virus (HRSV-S2) and human adenovirus serotype 5 (HAdV-5), in contrast to, for example, AH-V, where the activity was slightly reduced, and the only difference was the additional presence of an adamantane group in the structure of AH-V. However, the cytotoxicity of the compounds increases as follows: C-V < H-V < AH-V < AC-V < NCH7 < NH7C as in the last four compounds it is 10 and more times higher. It can be seen that the compounds with the highest cytotoxicity are those peptide derivatives containing adamantane (AC-V and AH-V) and niacin (NCH7 and NH7C). Antimicrobial tests showed promising activity of the newly synthesized compounds against the used Gram-positive and Gram-negative bacteria. The compounds C-V, H-V, AC-V, and AH-V were found slightly more active than NH7C and NCH7. 

In the study of the biological activity of the modified cotton material, a decrease in the antiviral activity of the peptide derivatives was observed, with compound H-V again giving the highest values. The antibacterial activity has been retained after the deposition of the compounds on cotton fabric. In conclusion, of all the compounds studied with the most promising results for potential application to enhance the protective properties against pathogens of cotton textiles is the H-V peptide, containing histidine amino acid residue.

## Figures and Tables

**Table 1:** Characteristics of the new synthetic antimicrobial peptides.

Peptide Code	Peptide Structure	Molecular Formula	^a^ [MH]^+^ ^Calculated^	^a^ [MH]^+^ ^Observed^	^b^ t_R, min_	^c^ [α]_546_^20^ (°)
C-V	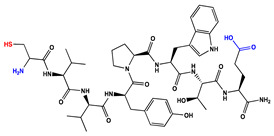	C_47_H_66_N_10_O_12_S	994,4582	995,4631	18.75	−54
H-V	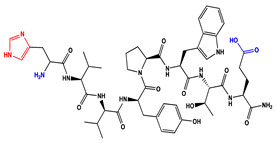	C_50_H_68_N_12_O_12_	1028,5080	1029,5148	16.85	−40
AC-V	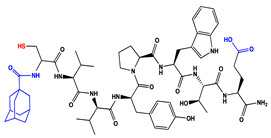	C_58_H_80_N_10_O_13_S	1156,5627	1157,5685	38.14	−44
AH-V	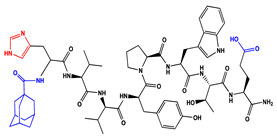	C_61_H_82_N_12_O_13_	1190,6124	1191,6147	29.26	−44
NH7C	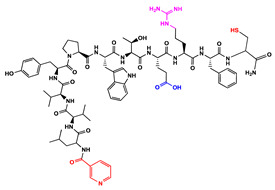	C_74_H_101_N_17_O_16_S	1515,7333	1516,7407	25.89	−34
NCH7	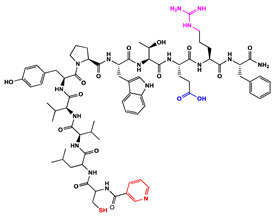	C_74_H_101_N_17_O_16_S	1515,7333	1516,7406	25.82	−36

^a^ The mass ion (MH^+^) was obtained by electrospray ionization mass spectrometry (ESI-MS); ^b^ **t**_R_ is the retention time determined by HPLC; ^c^ optical rotation in methanol (c = 0.01) at 20 °C.

**Table 2:** Spectral characterizations of test compounds in neutral solution.

Compounds	λ_abs_ [nm]	ε [L/(mol·cm)]	λ_em_ [nm]	Stokes Shift [cm^−1^]	Quantum Yield
AH-V	295	6.62 × 10^5^	356	5808	0.67
C-V	295	1.27 × 10^6^	357	5887	0.24
H-V	295	1.30 × 10^6^	356	5808	0.31
AC-V	295	6.12 × 10^5^	354	5650	0.61
NH7C	295	7.49 × 10^5^	355	5729	0.27
NC7H	295	1.44 × 10^5^	355	5729	0.12

**Table 3:** Virucidal effect of both new VV-hemorphin-5 and VV-hemorphin-7 analogues against human respiratory syncytial virus (HRSV-S2) and human adenovirus serotype 5 (HAdV-5) after 30 min/60 min.

Virus	Δlog 30 min						Δlog 60 min					
	C-V	H-V	AC-V	AH-V	NH7C	NCH7	C-V	H-V	AC-V	AH-V	NH7C	NCH7
HRSV-2	0.1	0.1	0.1	0.1	0.2	0.1	1.1	1.6	1.4	1.3	1.0	0.9
HAdV-5	0	0	0	0	0	0	0	0	0	0	0	0

**Table 4:** Virucidal effect of new cotton fabrics dyed with new hemorphin peptides against human respiratory syncytial virus (HRSV-S2) and human adenovirus C serotype 5 (HAdV-5) after 30 min/ 60 min.

Virus	Δlog 30 min						Δlog 60 min					
	C-V-Textile	H-V-Textile	AC-V-Textile	AH-V-Textile	NH7C-Textile	NCH7-Textile	C-V-Textile	H-V-Textile	AC-V-Textile	AH-V-Textile	NH7C-Textile	NCH7-Textile
HRSV-2	0	0	0	0	0	0	0.9	1.0	1.0	0.9	0.8	0.6
HAdV-5	0	0	0	0	0	0	0	0	0	0	0	0

**Table 5:** Cytotoxicity of new N- and C-modified peptide analogues of hemorphins in HEp-2 cell culture.

Compound	Cytotoxicity
	CC_50_ (µM/mL) in HEp-2 Cells
C-V	10.7
H-V	12.2
AC-V	110
AH-V	106
NH7C	170
NCH7	139
Ribavirin	2103

## Supplementary Materials

The following supporting information can be downloaded at: https://www.mdpi.com/article/10.3390/molecules27154770/s1. Figure S1. FTIR spectrum of investigated peptide derivatives (C-V, H-V, AC-V, AH-V, NH7C, and NCH7). Figure S2. Subtracting the FTIR spectrum of cotton fabric from the spectra of cotton fabric modified with AC-V, AH-V, C-V, H-V, NCH7, and NH7C. Figure S3. ESI-MS spectra of new peptide analogues (C-V, H-V, AC-V, AH-V, NH7C, and NCH7). Figure S4. Analytical HPLC chromatograms of peptides.

## Sample Availability

Samples of the compounds are available from the authors.
