# Supplementary material for: Study of Novel Peptides for Antimicrobial Protection in Solution and on Cotton Fabric
Source: Molecules. 2022 Jul 26;27(15):4770. doi: 10.3390/molecules27154770 (PMC9332204; doi:10.3390/molecules27154770)
Supplement: Supplementary file 1 [file molecules-27-04770-s001.zip › molecules-1798090-supplementary.pdf]

Supplementary material

Study of Novel Peptides for Antimicrobial Protection in Solution and on Cotton Fabric

Petar Todorov [1*], Stela Georgieva[2], Desislava Staneva [3], Petia Peneva [1],

Petar Grozdanov [4], Ivanka Nikolova [4], Evgenia Vasileva-Tonkova [4], Ivo Grabchev [5]

[1]*Department of Organic Chemistry, University of Chemical Technology and Metallurgy, 1756 Sofia, Bulgaria; petenceto_2@abv.bg*

[2]*Department of Analytical Chemistry, University of Chemical Technology and Metallurgy, 1756 Sofia, Bulgaria;st.georgieva@uctm.edu*

[3]*Department of Textile, Leathers and Fuels, University of Chemical Technology and Metallurgy, 1756 Sofia, Bulgaria; grabcheva@mail.bg*

[4]*The Stephan Angeloff Institute of Microbiology, Bulgarian Academy of Sciences, 1113 Sofia, Bulgaria; grozdanov@microbio.bas.bg (P.G.); inikolova@microbio.bas.bg (I.N.); evaston@yahoo.com (E.V.-T.)*

[5]*Faculty of Medicine, Sofia University "St. Kl. Ohridski", 1407 Sofia, Bulgaria; i.grabchev@chem.uni-sofia.bg*

** Correspondence: pepi_37@abv.bg; Tel.: +359-2-8163423*

Table of Contents: Page

1. FTIR spectra S2
2. Mass spectra of synthesized compounds S4
3. Analytical HPLC chromatograms of peptides S7

1. FTIR spectra

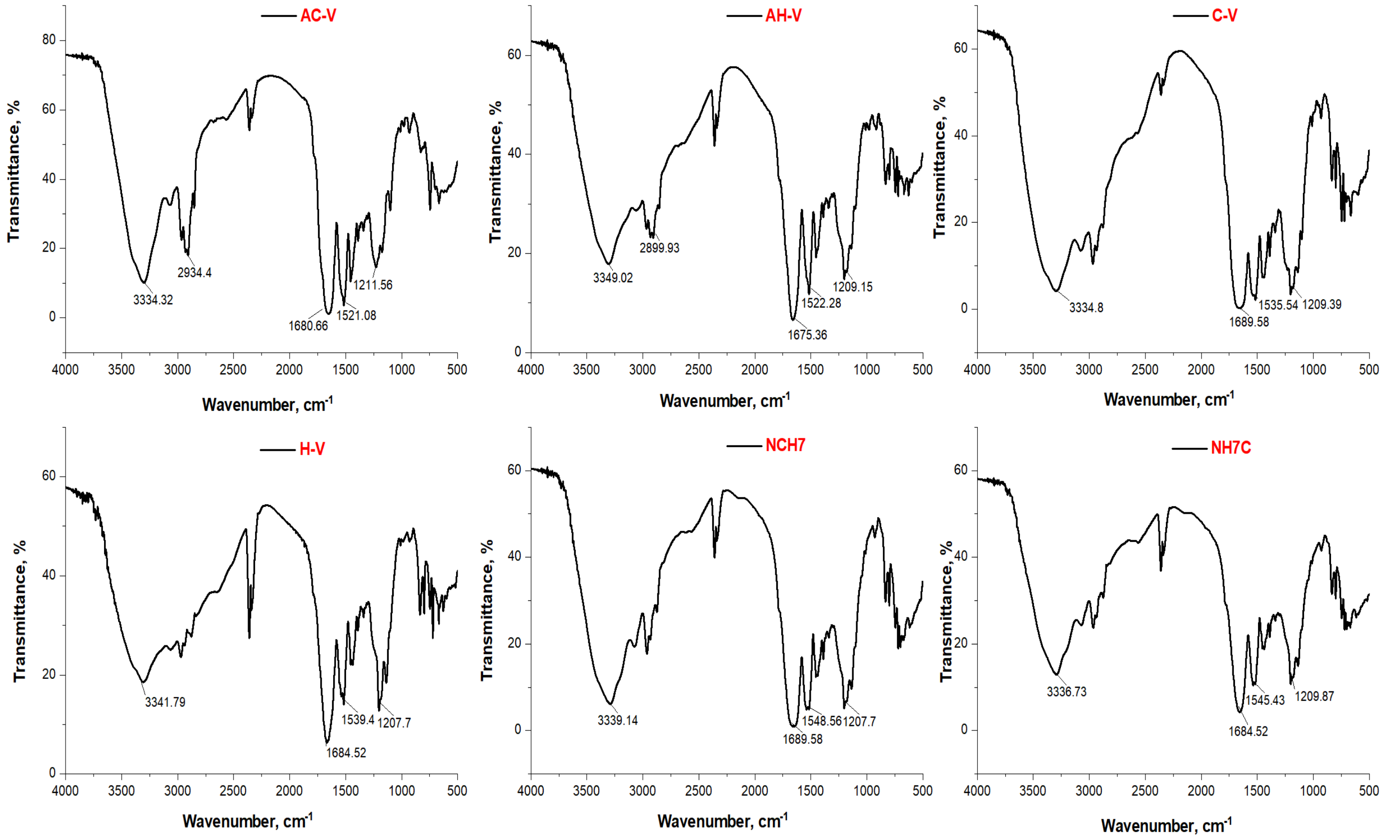

Figure S1. FTIR spectrum of investigated peptide derivatives (**C-V, H-V, AC-V, AH-V, NH7C** and **NCH7**)

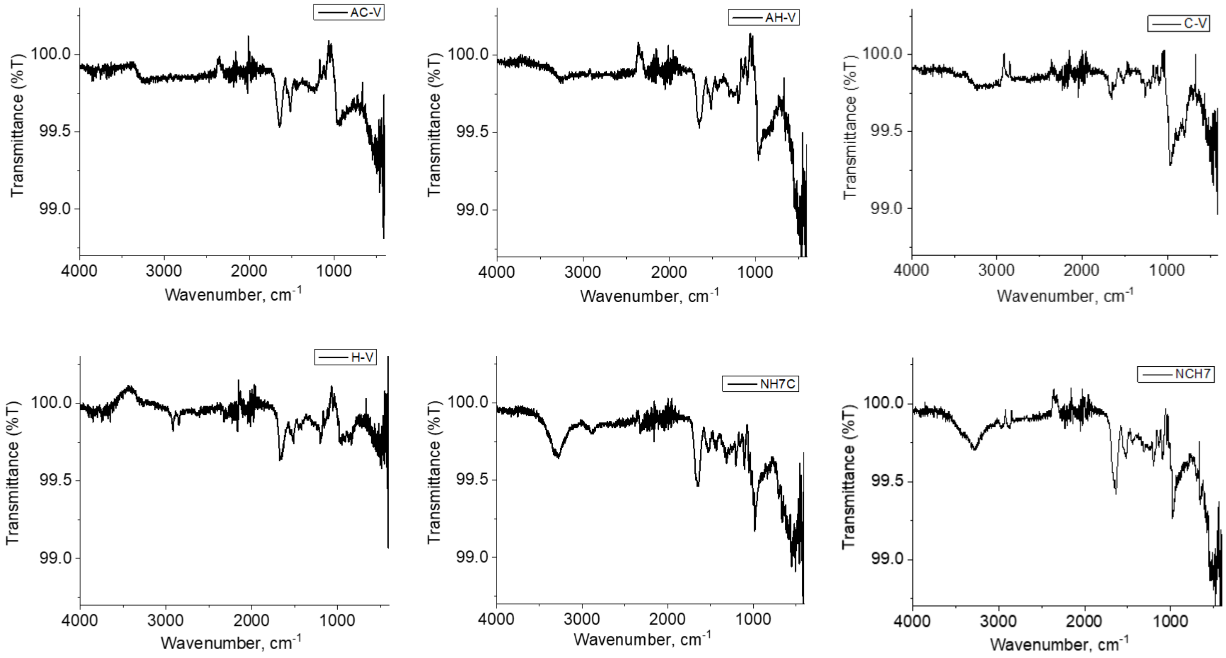

Figure S2. Subtracting FTIR spectrum of cotton fabric from the spectra of cotton fabric modified with **AC-V, AH-V, C-V, H-V, NCH7** and **NH7C**

2. Mass spectra of synthesized compounds

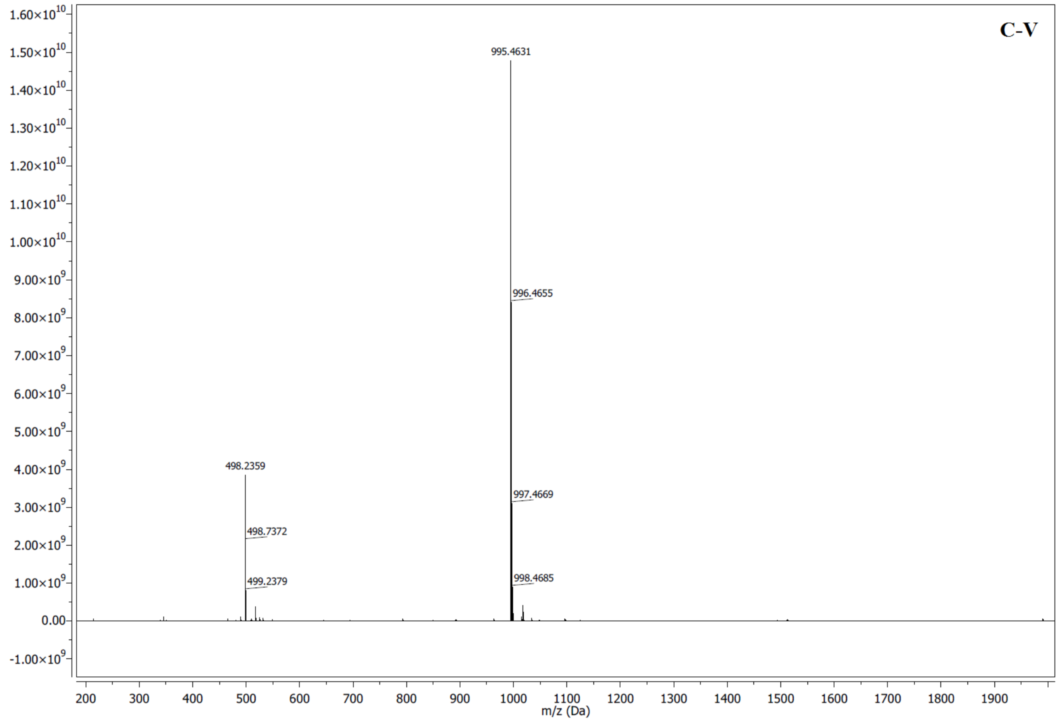

A) ESI-MS spectrum of C-V

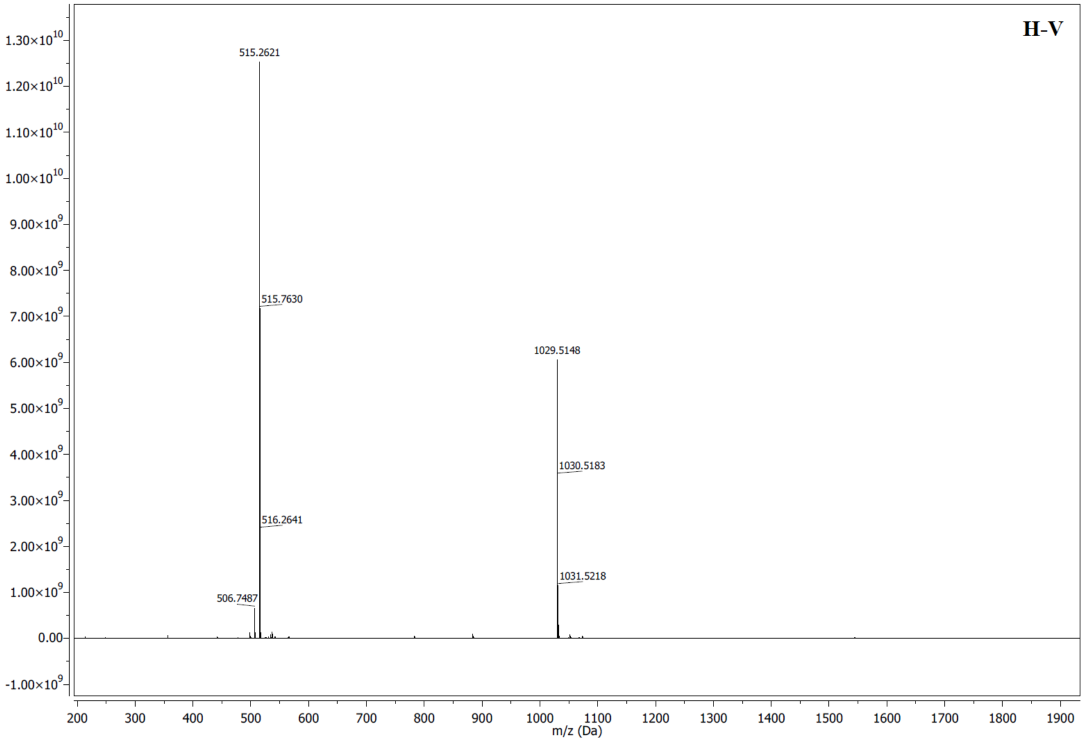

B) ESI-MS spectrum of H-V

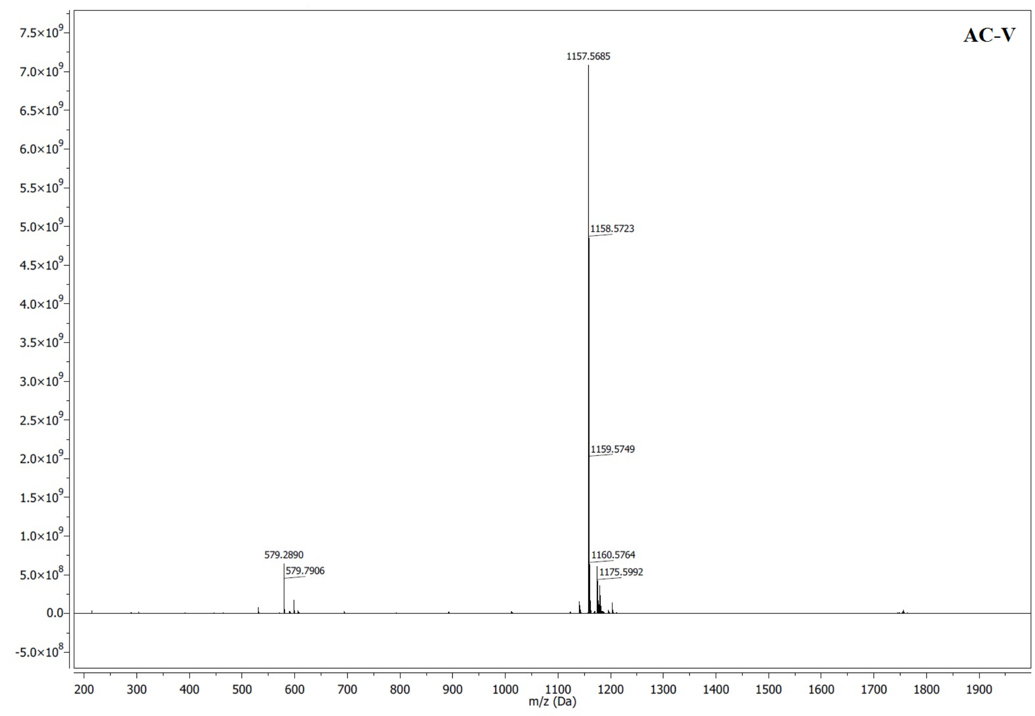

C) ESI-MS spectrum of AC-V

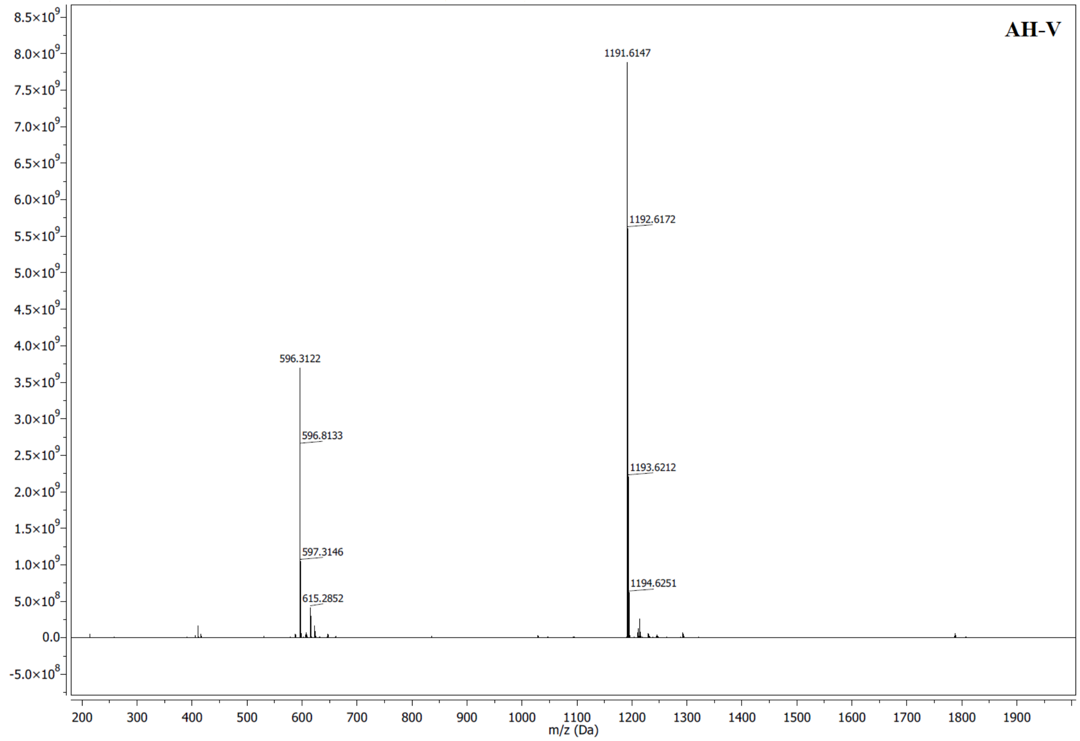

D) ESI-MS spectrum of AH-V

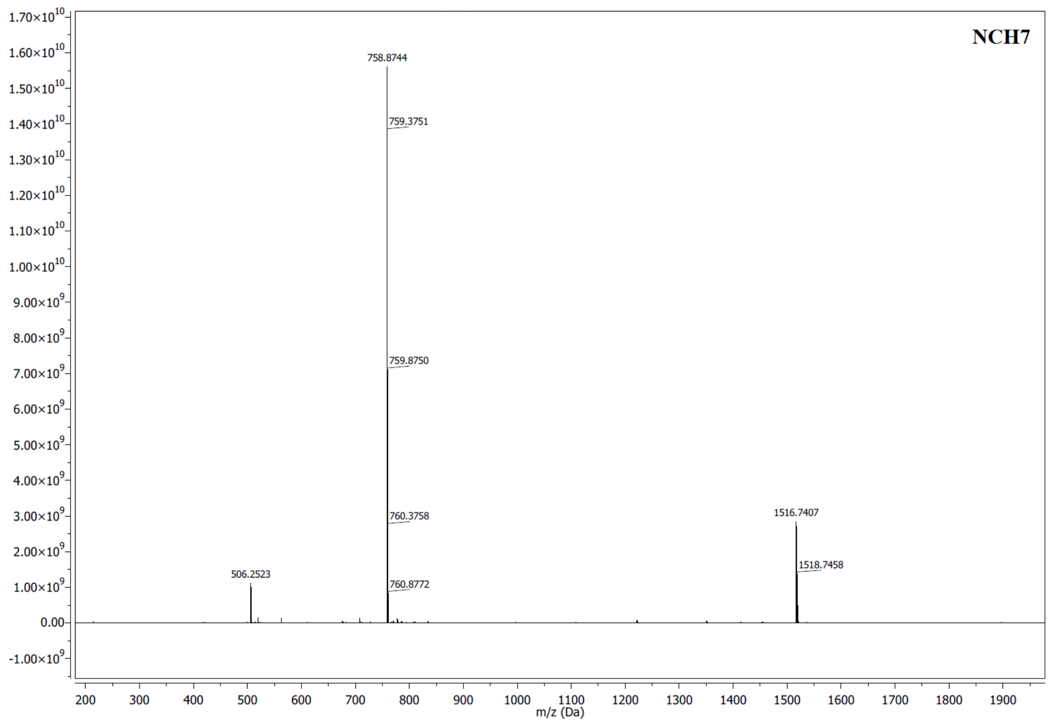

E) ESI-MS spectrum of NCH7

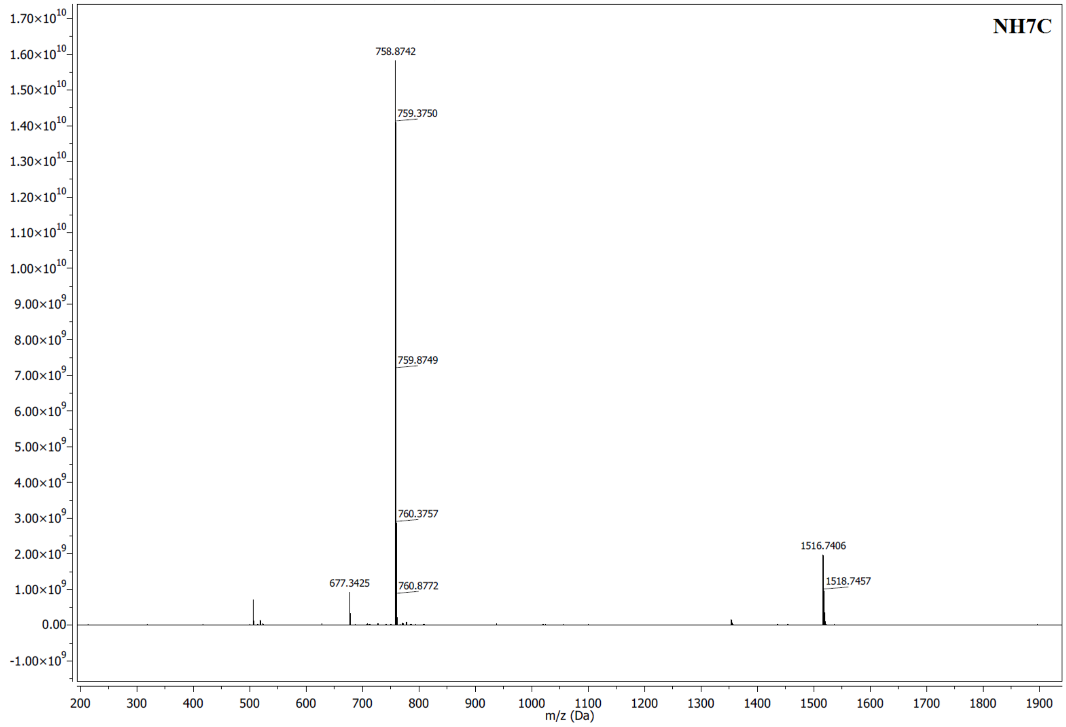

F) ESI-MS spectrum of NH7C

Figure S3. ESI-MS spectra of new peptide analogues (**C-V, H-V, AC-V, AH-V, NH7C** and **NCH7**)

3. Analytical HPLC chromatograms of peptides.

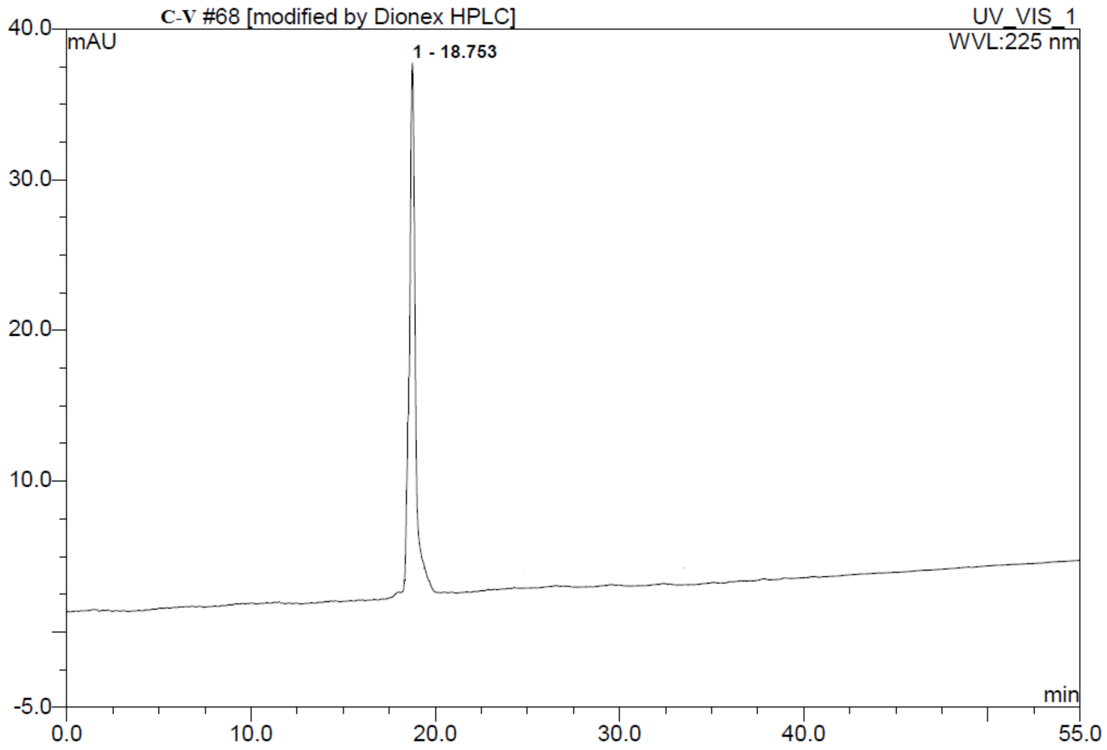

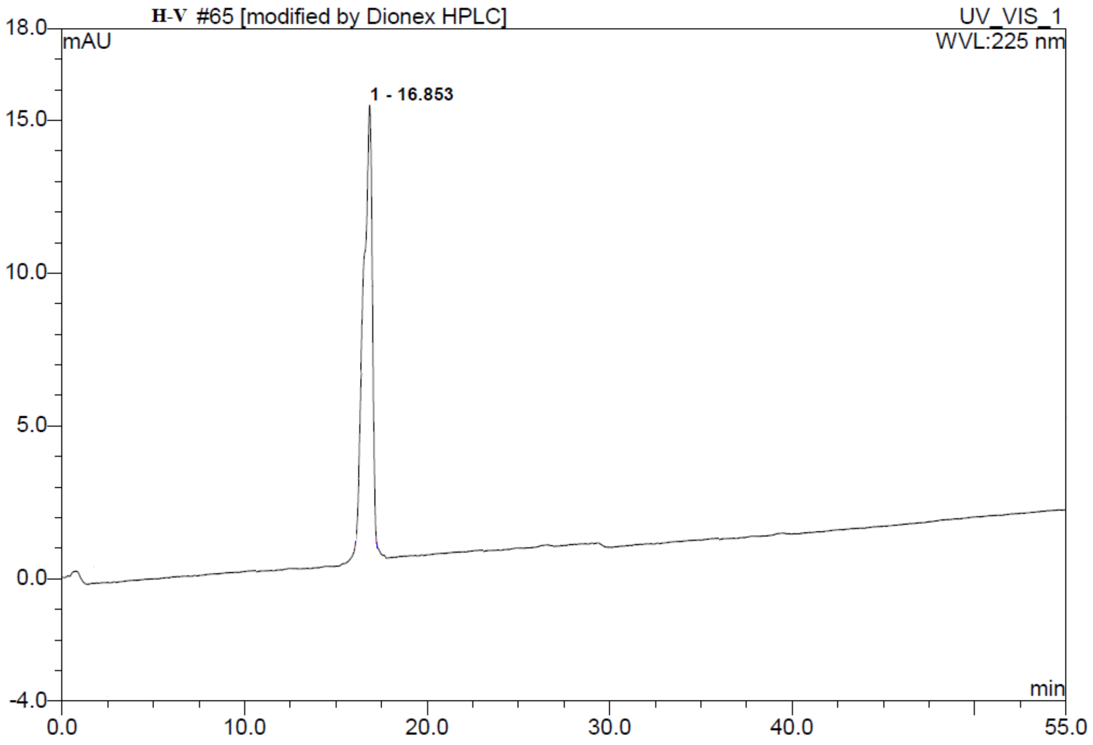

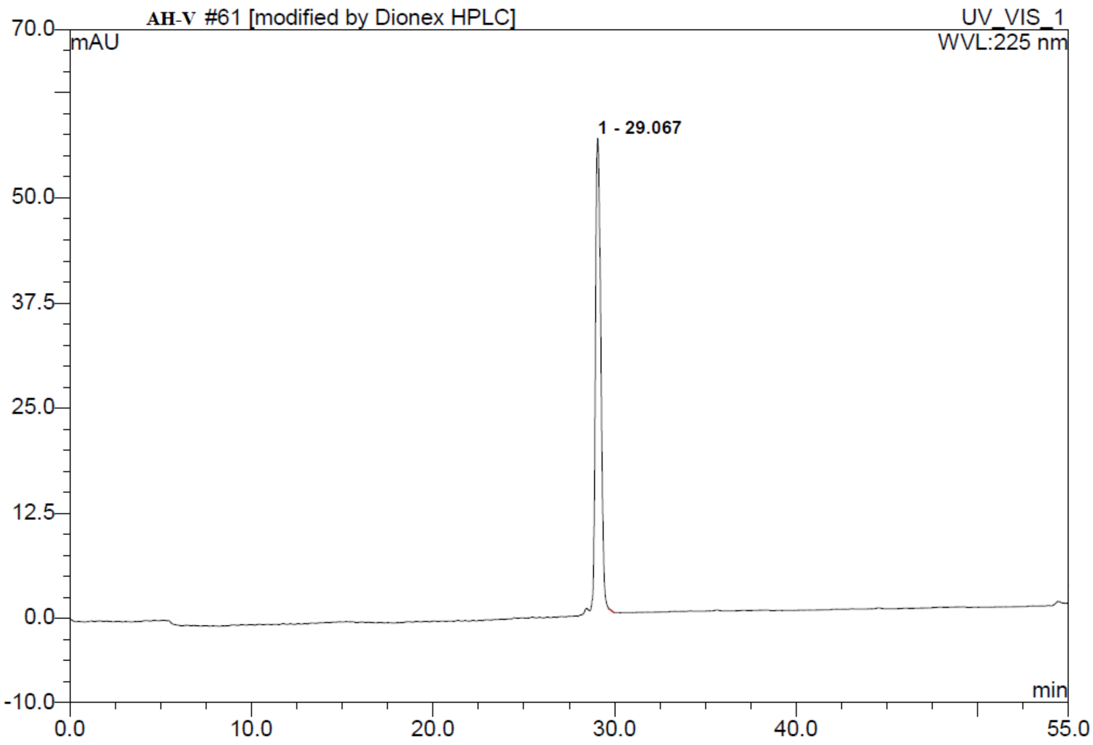
AH-V #61 [modified by Dionex HPLC]
UV_VIS_1
WVL:225 nm
mAU
1 - 29.067
70.0
50.0
37.5
25.0
12.5
0.0
-10.0
min
0.0
10.0
20.0
30.0
40.0
55.0

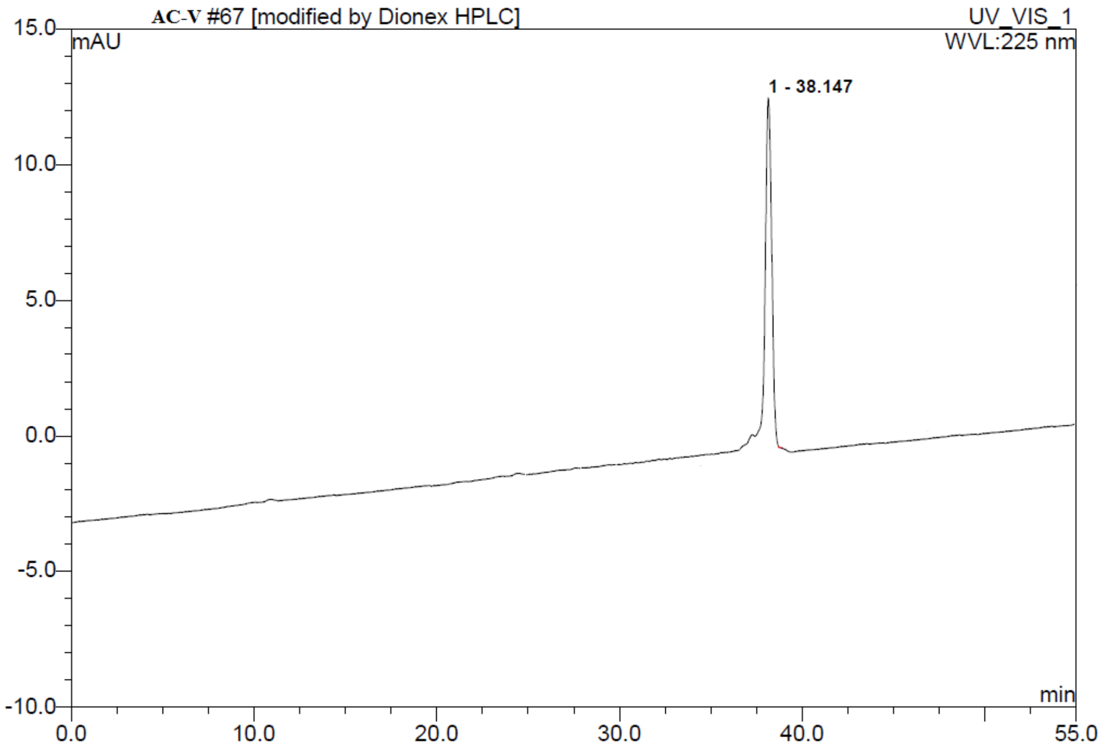
AC-V #67 [modified by Dionex HPLC]
UV_VIS_1
WVL:225 nm
mAU
1 - 38.147
15.0
10.0
5.0
0.0
-5.0
-10.0
min
0.0
10.0
20.0
30.0
40.0
55.0

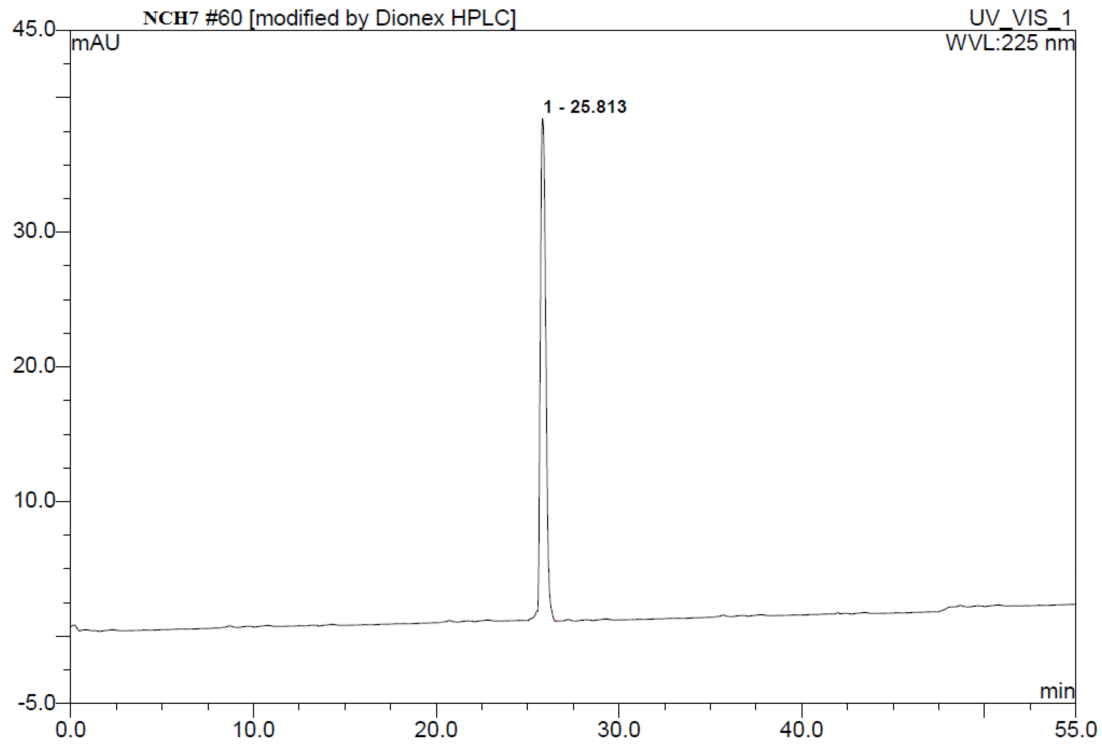

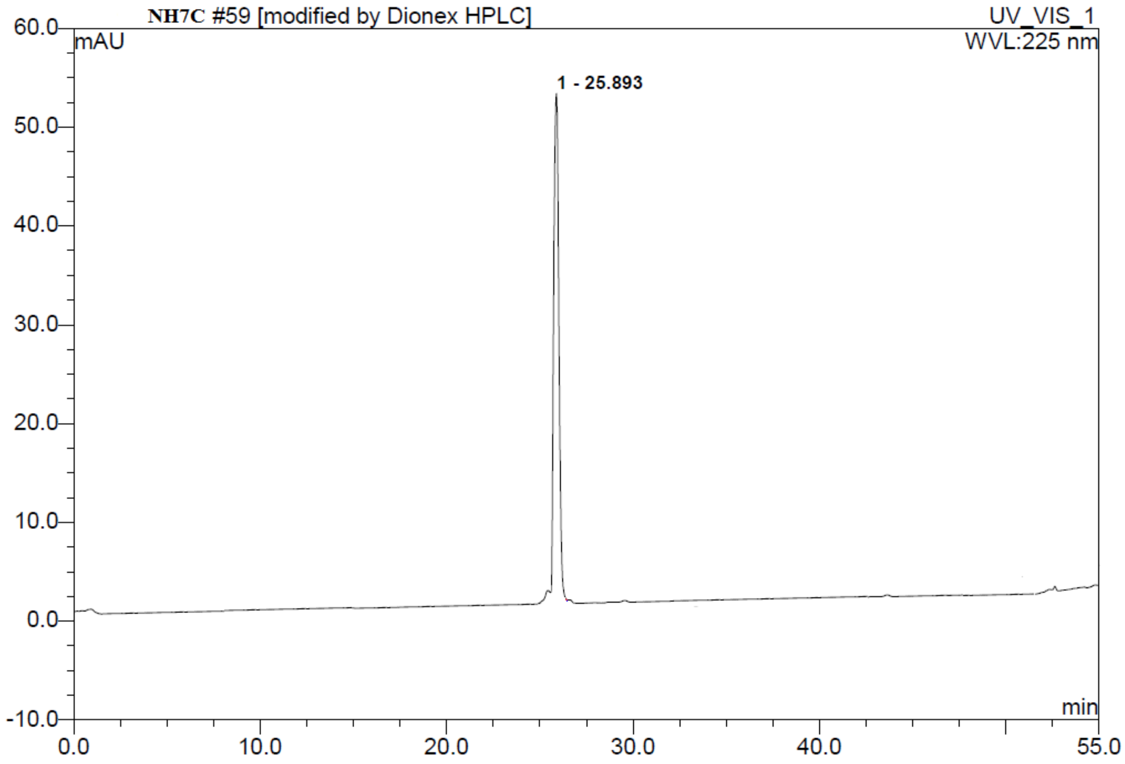

Figure S4. Analytical HPLC chromatograms of peptides
